# Laser-Driven Single-Step Synthesis of Monolithic Prelithiated Silicon-Graphene Anodes for Ultrahigh-Performance Zero-Decay Lithium-Ion Batteries

**DOI:** 10.1007/s40820-026-02074-2

**Published:** 2026-01-26

**Authors:** Avinash Kothuru, Gil Daffan, Fernando Patolsky

**Affiliations:** 1 School of Chemistry, Faculty of Exact Sciences, Tel Aviv University, 69978 Tel Aviv, Israel; 2 Department of Materials Science and Engineering, Faculty of Engineering, Tel Aviv University, 69978 Tel Aviv, Israel; 3 Tel Aviv University Center for Nanoscience and Nanotechnology, Tel Aviv University, 69978 Tel Aviv, Israel

**Keywords:** Laser-induced graphene, Prelithiation, Silicon anode, Lithium-ion battery, Artificial SEI

## Abstract

Silicon-based anodes offer a promising alternative to graphite in lithium-ion batteries (LIBs) due to significantly higher energy density. However, their practical application is limited by substantial volume expansion during lithiation, which causes structural instability and continuous formation of the solid electrolyte interphase (SEI), drastically reducing initial coulombic efficiency (ICE) and capacity retention. Strategies such as silicon nanostructuring and integration with conductive carbon matrices help accommodate volume changes and improve conductivity but fall short in fully addressing lithium loss and long-term capacity fade. Prelithiation can mitigate these issues by compensating for lithium loss and stabilizing the SEI. However, conventional prelithiation methods are complex, air-sensitive, multi-step, and ex situ, often requiring reactive lithium metal or exotic lithium salt precursors. In response, this study introduces a laser-driven, solid-state, ambient, in situ prelithiation method performed concurrently with the synthesis of silicon-graphene pseudo-monolithic composite anodes. A ternary blend of phenolic resin, silicon nanoparticles (SiNPs), and common lithium salts, subjected to rapid, low-power laser irradiation, produces a self-standing, air-stable, prelithiated composite, where the resulting porous and conductive matrix encapsulates the SiNPs, while the unique laser-induced environment triggers in situ reactions that prelithiate the silicon surface and form stable covalent interfaces. The resulting lithiated anodes reveal remarkable features, delivering over 1700 mAh g^−1^ with negligible capacity decay (< 2%) over 2000 + cycles at 5 A g^−1^, 83% retention after 4500 cycles, and ICE above 97% versus non-lithiated counterparts. The anodes also display ultrafast charging capabilities, retaining up to 63% of their maximum capacity at 10 A g^−1^. This innovation not only advances the development of next-generation LIBs, but also establishes a framework for converting readily available and cost-effective precursor materials into high-performing electrodes, promising to reduce complexity and costs in battery manufacturing.

**Supplementary Information:**

The online version contains supplementary material available at 10.1007/s40820-026-02074-2.

## Highlights

- We report an ambient single-step laser-driven process that simultaneously synthesizes and integrates prelithiated silicon nanoparticles into a robust graphene matrix using simple precursors.
- Prelithiation is achieved in situ through interfacial solid-state reactions between Si and common lithium salt precursors during the ultrafast photothermal graphitization of phenolic resin.
- Prelithiated silicon nanoparticles/laser-induced graphene anodes exhibit exceptional cycling stability (> 98% capacity retention after 2000 cycles) and near-zero performance decay in Li-ion half and full cells compared to non-lithiated counterparts.

**Supplementary Information:**

The online version contains supplementary material available at 10.1007/s40820-026-02074-2.

## Graphical abstract

**Figure:**
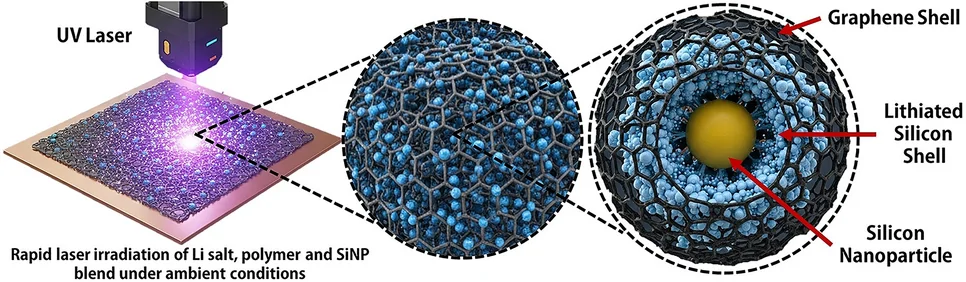

## Introduction

Lithium-ion batteries (LIBs) power technologies ranging from portable electronics to electric vehicles and grid storage. Their high energy density, long cycle life, and low self-discharge make them the preferred choice for many applications. As energy storage demand grows, enhancing LIB performance for higher energy density, faster charging, and longer lifespan is crucial [1]. A key factor in this advancement is the anode material, with graphite being the most common. While cost-effective and stable, its low theoretical capacity (372 mAh g^−1^) limits further improvements in energy density and performance [2].

Silicon has attracted significant research attention in recent years as a promising anode material [3–10], owing to its high theoretical capacity (3579 mAh g^−1^), far exceeding that of graphite [11]. However, its practical application is hindered by severe volume expansion (up to 400%) during lithiation, which induces mechanical stress and leads to material pulverization, thus compromising electrical contact and capacity retention. Additionally, repeated fracture and reformation of the solid electrolyte interphase (SEI) result in continuous lithium-ion and electrolyte consumption, severely impacting coulombic efficiency (CE) and cycle life, thereby hindering the viability of silicon-based anodes in LIBs [12].

Beyond these intrinsic challenges, several strategies have been explored to enhance the performance of silicon-based anodes. Nanostructuring and porous silicon architectures help mitigate volume expansion by accommodating mechanical strain, while carbon composites improve electrical conductivity and provide structural support. These approaches have led to notable gains in capacity retention and cycling performance. However, long-term stability remains a concern, and many of these methods face limitations in scalability and practical implementation. This highlights the continued need for alternative or complementary solutions.

Prelithiation is another promising strategy to compensate for initial lithium loss in silicon anodes, addressing high irreversible capacity loss and repeated SEI growth [13, 14]. By pre-loading lithium before cell assembly, it can enhance initial coulombic efficiency (ICE) and mitigate early cycle lithium and electrolyte depletion, leading to improved capacity, cycle life, and overall battery performance [15, 16].

Yet, despite its promise, current prelithiation techniques face substantial challenges, particularly in effective synthesis and scalability [17, 18]. Direct contact prelithiation, where lithium metal is mechanically applied directly to the anode and chemically introduced, is somewhat effective but fraught with practical difficulties due to the extreme reactivity of lithium metal [19]. Safe handling requires a controlled, inert atmosphere, increasing complexity and cost. Moreover, achieving uniform prelithiation across the anode remains challenging, often resulting in inconsistent performance.

Chemical prelithiation methods offer precise control but face significant challenges in scalability and practicality. Typically involving wet or gas-phase reactions, these methods also frequently require high temperatures and controlled atmospheres, making them potentially energy-intensive and costly [20]. Furthermore, the complex multi-step nature of these processes often results in prolonged reaction times to achieve sufficient prelithiation. For instance, lithium-organic compounds like lithium biphenyl (Li-BP) effectively prelithiate silicon anodes, but the process demands strict temperature control and extended exposure to ensure uniform lithium distribution, sometimes taking hours. Moreover, these techniques often rely on expensive and scarce lithium salts, increasing material costs and hindering large-scale production.

Thus, while prelithiation offers a significant opportunity to enhance silicon anode performance, the development of more rapid, cost-effective, and air-stable techniques using common and non-hazardous lithium precursors is essential for its commercial success.

In response to these challenges, this work introduces a different approach to prelithiation through a single-step, low-energy, ambient, solid-state, and additive-free process triggered by laser-induced graphene (LIG) formation. LIG is a versatile material synthesized by rapidly transforming commercial polymers or even biomass into defect-rich, porous, and three-dimensional graphene structures using low-power laser irradiation ranging from ultraviolet to infrared wavelengths [21]. This process generates extreme localized temperatures (> 2000 K) and pressures (> 1 GPa) [22, 23] inducing the rearrangement of carbon networks into graphene nanodomains. Due to its rapid formation capability, low-energy input requirements, and ability to be synthesized under ambient conditions, LIG has emerged as a promising platform for energy storage, catalysis, and sensing applications.

Building on this foundation, recently reported in situ functionalization strategies leveraged the unique thermodynamic conditions formed in the LIG synthesis microenvironment to drive in situ chemical transformations, enabling the single-step fabrication of functional, self-standing, and additive-free composite materials for energy applications [24–28]. Accordingly, this method facilitates the integration and/or synthesis of active materials directly into the as-forming conductive LIG matrix, eliminating the need for multi-step post-processing and creating a mechanically robust pseudo-monolithic structure.

Previously, the first demonstration of this approach utilized a blend of silicon nanoparticles (SiNPs) and LIG precursors, where the as-forming conductive LIG matrix encapsulated the SiNPs, yielding stable, high-performance composite anodes for LIBs [24, 25]. The high surface area and conductive LIG matrix embedded with SiNPs facilitated efficient electron and ion transport, enhancing electrochemical performance. Additionally, its porous architecture effectively accommodated silicon’s volume expansion during lithiation, reducing mechanical stress and significantly improving long-term cycling durability. Furthermore, this approach has been shown to enable the in situ sublimation of elements such as phosphorus and sulfur, followed by the covalent integration of molecular adducts into the forming LIG matrix [26, 27]. This strategy has also demonstrated versatility in directly reducing and encapsulating metal nanoparticles from precursor salts, further broadening its applicability in functional material synthesis [28].

In this work, we advance this approach by leveraging the unique microenvironment of LIG synthesis to trigger the in situ prelithiation of an air-stable, additive-free, and self-standing SiNP-LIG composite. By utilizing a ternary blend of a LIG polymer precursor, SiNPs, and a common lithium salt (e.g., hydroxide, carbonate, etc.), laser-triggered in situ chemical reactions are induced, rapidly prelithiating the SiNP surface under ambient conditions and also forming interfacial silicon carbides. The LIG matrix encapsulates the lithiated silicon in a high surface area and porous matrix, ultimately resulting in a ready-to-assemble anode with no need for post-processing, binders, or conductive additives.

Advanced characterization techniques, including electron energy loss spectroscopy (EELS) and time-of-flight secondary ion mass spectroscopy (TOF–SIMS), depict the chemical transformation of lithium and its distribution within the composite. This prelithiation strategy dramatically enhances ICE ( > 97%) and lithium availability, delivering exceptional stability with virtually no capacity decay (> 98% retention) after 2000 cycles at high current densities (5 A g^−1^), while maintaining a high gravimetric capacity of over 1700 mAh g^−1^, far surpassing identical anodes cycled without prelithiation and outperforming many previously reported state-of-the-art prelithiated silicon-based anodes. Additionally, extensive electrochemical analyses demonstrated a substantial increase in lithium diffusion properties, significantly boosting ion transport kinetics and rate performance. Thus, this remarkable improvement over its non-lithiated counterpart highlights the critical role of in situ prelithiation in not only stabilizing the anode but also maximizing high-power capability.

Herein, by integrating laser graphitization-driven in situ prelithiation into the synthesis of silicon-graphene composites, we introduce a new prelithiation approach to overcoming key limitations in silicon anode performance. Beyond enhancing electrochemical performance, this method represents a paradigm shift in simultaneous silicon anode fabrication and prelithiation strategies. Unlike conventional techniques that require complex multi-step processing, hazardous lithium precursors, or controlled environments, this approach enables rapid, low-energy, ambient, and direct prelithiation using common lithium salts, achieved simultaneously within the same step as host composite synthesis. This innovation significantly advances the practicality of prelithiation, bringing it closer to real-world implementation in next-generation high-performance LIBs.

## Experimental

### Anode Synthesis

A solution of phenol–formaldehyde resin (PR, Polyols & Polymers PVT LTD) in anhydrous ethanol (99.9%) was prepared by dissolving 5 g of PR in 10 mL of ethanol under magnetic stirring until full dissolution. To achieve specific Si:Li mass ratios of 1:0, 3:1, 1:1, and 2:1, appropriate amounts of LiOH (Sigma-Aldrich) were first dissolved in the phenolic resin solution. Subsequently, 0.3 g of silicon nanoparticles (SiNPs, Nanografi, 100 ± 20 nm) were dispersed into this solution to form the prelithiation slurry. Control anodes were prepared using the same procedure but without the addition of any lithium salt.

To investigate the effect of lithium salt type on the prelithiation process, Li_2_CO_3_, LiF, LiNO_3_, and LiClO_4_ (Sigma-Aldrich) were each incorporated at an equivalent 1:1 Li molar ratio relative to the 1:1 Si:LiOH mass ratio for consistent comparison. All slurries were ultrasonicated in an ice bath for 20 min to prevent agglomeration and ensure uniform dispersion.

For anode preparation, approximately 100 µL of the slurry was deposited per coating cycle onto copper-foil current collectors (12 µm thickness, 99.9% Cu) using a programmable spin coater operated at 500 rpm for 10 s and 3000 rpm for 30 s. Each layer was prebaked at 40 °C for 5 min and 60 °C for 25 min. Three sequential coatings produced a uniform composite film with a total thickness of ~ 50 µm (measured by profilometry).

Laser-driven conversion and in situ prelithiation were performed under ambient atmosphere using a continuous-wave 450 nm diode laser (Zmorph Fab, 2.8 W output). The beam (~ 50 µm spot diameter) was rastered across the surface at 100% power, a scan speed of 20 mm s^−1^, hatch spacing 0.1 mm, and defocusing height 5 mm. These parameters were previously optimized in our earlier works [24, 26] to ensure efficient carbonization, uniform lithium incorporation, and optimal electrochemical performance. No binders or conductive additives were used in any step, and the anode mass loading optimized at approximately 1 mg cm^−2^.

### Material Characterization

High-resolution scanning electron microscopy (HR-SEM) imaging and energy-dispersive X-ray spectroscopy (EDS, Bruker XFlash 6/60) were conducted using a Zeiss GeminiSEM 300 system. Transmission electron microscopy (TEM), scanning transmission electron microscopy (STEM), electron energy loss spectroscopy (EELS), and further EDS analyses were conducted using an aberration-corrected Themis Z G3 system (Thermo Fisher). Cross-sectional imaging and lamella preparation for TEM analysis were performed using a Helios Nanolab 460F1Lite Dual Beam Focused Ion Beam/Scanning Electron Microscope (FIB/SEM, Thermo Fisher).

X-ray photoelectron spectroscopy (XPS) measurements were conducted using an ESCALAB QXi X-ray Photoelectron Spectrometer Microprobe with an Al K*α* source. Post-mortem XPS battery analysis was conducted after delithiation using an inert chamber module that can be sealed in a glove box under an inert atmosphere and opened within the XPS machine.

Raman spectroscopy measurements were performed using a LabRAM Soleil Confocal micro-Raman (Horiba, France) with a 532 nm laser. Time-of-flight secondary ion mass spectrometry (TOF-SIMS) was conducted using a Model TRIFT97 system (Physical Electronics) using the negative ion mass spectrum.

Powder X-ray diffraction (PXRD) analysis was conducted using a Bruker D8 Discover diffractometer with a Cu K*α* source. Lattice spacing using relevant peaks was calculated using Bragg’s law:

1 $$d = \frac{n\lambda }{{2\sin \theta }}$$

where *d* (Å) is the lattice spacing, *n* is the diffraction order, *λ* (Å) is the X-ray source wavelength (1.54 Å), and *θ* (º) is the glancing angle.

Additionally, crystallite size was estimated using the Scherrer equation:

2 $$D = \frac{K\lambda }{{\beta \cos \theta }}$$

where *D* (nm) is the average crystallite size, *K* is the shape factor (~ 0.9 is the typical value for crystallites with undefined geometry [29]), *λ* (Å) is the X-ray wavelength (1.54 Å), *β* (radians) is the full-width at half maximum (FWHM) of the diffraction peak, and *θ* (º) is the Bragg angle.

For the graphene component, the stacking height (*L*_*c*_)was calculated using the FWHM of the characteristic (002) reflection, as this peak corresponds to the diffraction from the stacked graphene layers along the c-axis. Conversely, the in-plane crystallite size (*L*_*a*_) was derived from the (100) reflection, which represents the diffraction from the hexagonal carbon network within the basal plane.

Inductively coupled plasma mass spectrometry (ICP-MS) was conducted using an Agilent 7800 ICP-MS system. The specific surface area was measured through Brunauer–Emmett–Teller (BET) analysis using a TriStar II Plus System (Micromeritics) with N_2_ gas as the adsorbate. Pore size distribution and volume were analyzed using the Barrett–Joyner–Halenda technique within the equipment software.

### Electrochemical Measurements

Half-cells were assembled as CR2032 coin cells. The cells containing the dried anode (12 mm diameter) pre-deposited on a current collector (12 μm thick copper foil), separator, lithium metal, 316L stainless steel spacer (15 mm diameter, 0.5 mm thickness) and a spring were assembled in a glovebox (< 0.1 ppm O_2_/H_2_O). The separator used was a 2325 Celgard separator, and the lithium metal disks (15 mm diameter) were purchased from S4R, France. The electrolyte used in all experiments was 50 μL of 85% 1 M LiPF_6_ in EC/DEC (1:1) and 15% fluoroethylene carbonate (FEC, Solvay-Fluor). Full cells were assembled identically but with lithium iron phosphate (LiFePO_4_, LFP, Nanografi) cathodes inserted.

The cathodes for the full cells were prepared by making a slurry of LFP (Nanografi), polyvinylidene fluoride (PVDF, Nanografi), and carbon black (Super P, Nanografi) in N-methyl-2-pyrrolidone (NMP) solvent at a mass ratio of 90:5:5. The slurry was cast accordingly, resulting in a final cathode loading of ~ 1 mg cm^−2^.

Galvanostatic cycling with potential limitation (GCPL) was conducted using a Neware BTS4000 battery cycler at a voltage range of 0.05–1 V versus Li/Li^+^. Full cells were cycled between 2.5 and 3.8 V. Post-mortem analysis was conducted after delithiation.

Cyclic voltammetry (CV) and potentiostatic electrical impedance spectroscopy (PEIS) measurements were conducted using a Bio-Logic BCS battery cycler. PEIS measurements were conducted using a frequency range of 0.01 MHz to 0.1 Hz with an amplitude of 10 mV after de-lithiation. EIS fitting was conducted using the Bio-Logic BT-Lab software, and the equivalent circuit used is detailed in Fig. S3. All cells were kept at 25 °C throughout all electrochemical experiments.

The Warburg element was calculated from the EIS data using the following equation [30]:

3 $$- Z_{im} = \sigma_{w} \omega^{{ - \frac{1}{2}}}$$

where − *Z*_*im*_ (Ohm) is the imaginary part of the impedance, *σ*_w_ is the Warburg constant (Ω s^−1.5^), and *ω* is the angular frequency (Hz) in the low frequency region.

The diffusion coefficient was further calculated using the following equation [30]:

4 $$D_{Li} = \frac{{R^{2} T^{2} }}{{2A^{2} n^{4} F^{4} C^{2} \sigma_{w}^{2} }}$$

where *D*_Li_ is the diffusion coefficient (cm^2^ s^−1^), *R* is the gas constant (kg m^2^ s^−2^ K^−1^ mol^−1^), *T* is the battery operation temperature (*K*), *F* is the Faraday constant (C mol^−1^), *A* is the electrode area (cm^2^), *C* is the lithium concentration in the electrolyte (mol cm^−3^), and *n* is the number of electrons transferred in the process.

## Results and Discussion

### Synthesis Procedure and Mechanism Overview

**Fig. 1:**
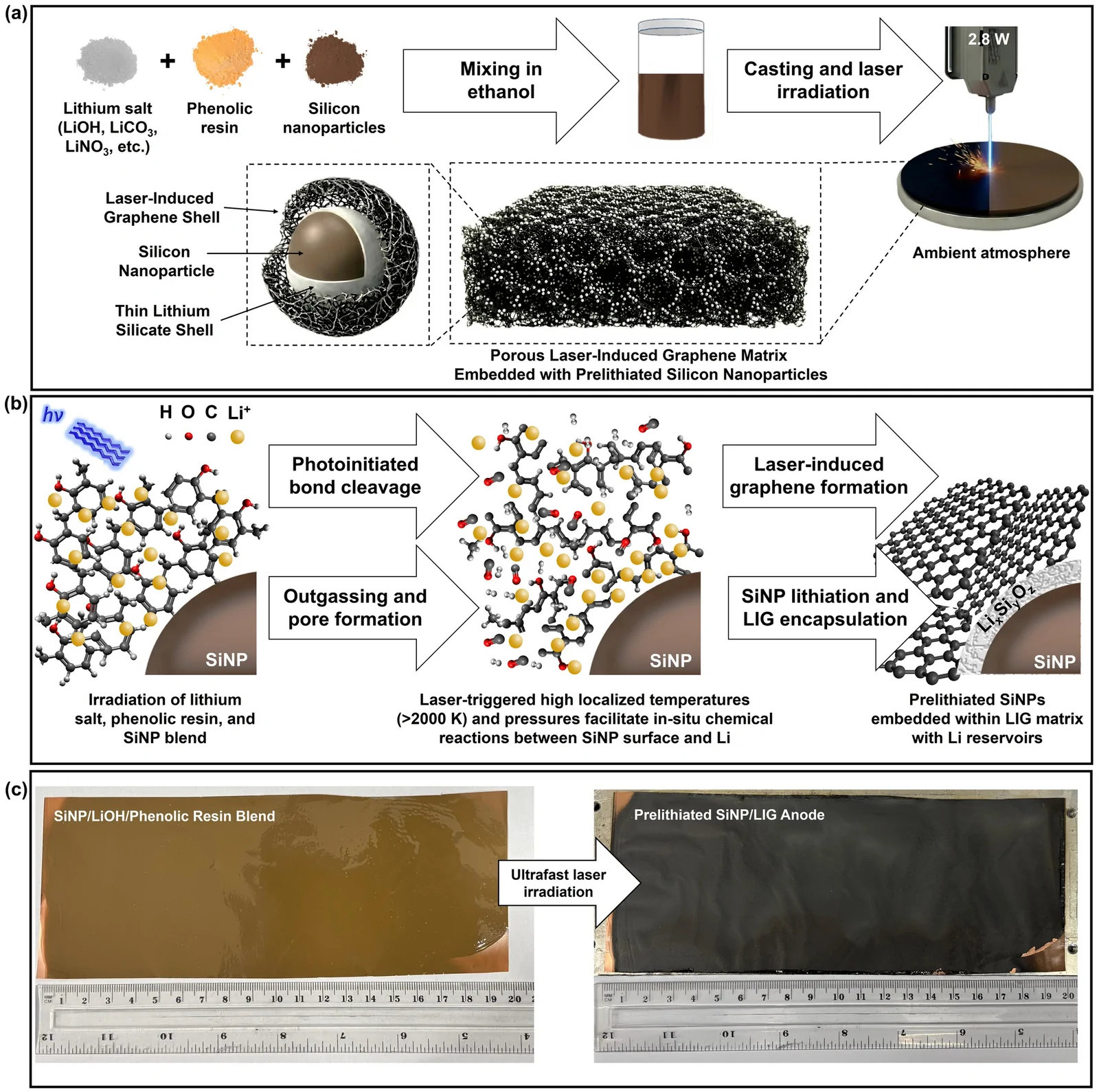
**a** Schematic overview of the single-step, ambient, and low-power laser irradiation process applied to a blend of Li salt, phenolic resin, and SiNPs for the synthesis of self-standing, porous, prelithiated PL-SiNP/LIG composite anodes. **b** Molecular-scale schematic with the proposed laser irradiation mechanism of the ternary blend, inducing LIG formation while concomitantly triggers in situ prelithiation and encapsulation of SiNPs. **c** Demonstration of prelithiated SiNP/LIG anode synthesis with large-area sheet formation, highlighting the scalability of the process

Figure 1a illustrates a schematic representation of the synthesis process. Initially, a solution consisting of anhydrous ethanol and phenol–formaldehyde resin was prepared. Phenolic resin was selected as the carbon precursor because it is a well-established material for LIG formation, yielding highly conductive and uniform graphenic films with excellent mechanical stability and electrochemical performance in energy-storage devices [24, 26, 28, 31–33]. It can be easily dissolved in inexpensive, non-hazardous polar solvents such as ethanol, forming a homogeneous reactive matrix that uniformly incorporates silicon nanoparticles and lithium salts, an advantage over common LIG precursors like polyimide, which is less soluble and less suited for in situ composite formation. Although other precursors such as sugars, asphalt, or natural polymers including cellulose, lignin, and collagen have been used for LIG synthesis, they typically require flame-retardant or dopant additives to prevent combustion and achieve partial graphitization, resulting in lower conductivity and smaller graphitic domains [34]. Phenolic resin therefore offers a practical and high-performance route for producing conductive, structurally stable graphene under ambient laser-processing conditions.

Ethanol was selected as a solvent for its non-hazardous and environmentally friendly characteristics, standing in stark contrast to commonly used hazardous solvents like N‐methyl‐2‐pyrrolidone (NMP) in battery fabrication. This choice, made in line with green chemistry principles, ultimately enhances the sustainability of the synthesis process.

Next, common lithium salts such as lithium hydroxide (LiOH), lithium nitrate (LiNO_3_), lithium perchlorate (LiClO_4_), lithium fluoride (LiF), or lithium carbonate (Li_2_CO_3_) were added to the solution and thoroughly dissolved to achieve a homogeneous mixture. Notably, this approach universally utilizes common Li salts, distinguishing it from nearly all reported prelithiation strategies that rely on more problematic Li precursors, such as highly reactive Li metal or exotic salts like Li-BP, which pose challenges related to handling, availability, stability, and scalability. The full dissolution of the resin and lithium salt ensures molecular-level dispersion and homogeneity of the reactive components, leaving the SiNPs as the only suspended solid in the slurry, an essential condition for achieving uniform reaction homogeneity during processing.

Subsequently, SiNPs with an average diameter of approximately 100 nm were introduced into the solution at varying mass ratios relative to the added Li salt, forming a slurry. The impact of varying silicon-to-lithium mass ratios (Si:Li) in the blend, as well as the effect of different precursor salts, will be explored in detail later in the manuscript. While all tested Si:Li ratios and salt types enhance performance to some extent, some prove to be more effective than others.

Then, to ensure uniform dispersion and prevent particle aggregation, the slurry underwent thorough mixing and probe sonication. The homogenized slurry was then deposited onto a current collector substrate (Cu foil) using conventional slurry deposition methods and subsequently dried. Depending on the specific application requirements, spin coating can be used for thin layers, while doctor blading can be employed for thicker films.

Optimization of laser parameters, including power, scanning speed, and defocusing height, was conducted as a function of the deposited film thickness to achieve optimal LIG quality and ensure precise irradiation depth control. These optimizations were carried out in accordance with previously published work using the same laser setup, precursor materials, and synthesis protocol, but without Li salt addition [24]. The inclusion of irradiation depth control is particularly critical to ensure a complete transformation of the precursor into LIG, preventing the presence of any residual insulating polymer layer between the anode and the current collector, which could otherwise hinder electrical conductivity and electrochemical performance.

Following film deposition and drying, the precursor film was subjected to low-power (2.8 W), rapid (> 20 mm s^−1^), visible (450 nm) laser irradiation and rastering under ambient conditions. Overall, this simple process results in a self-standing, additive-free, air-stable, and prelithiated SiNP/LIG (PL-SiNP/LIG) composite anode synthesized directly on the current collector substrate. The resulting composite anode is immediately ready for battery assembly without the need for any further post-processing, binders, or conductive additives, which are ubiquitously used in conventional electrode fabrication.

Figure 1b schematically illustrates the proposed molecular-scale reaction mechanism occurring within the precursor structure during laser irradiation, which will be further supported through rigorous structural and morphological characterization. Firstly, upon laser exposure, the well-established LIG formation mechanism is initiated [35]. The phenolic resin readily absorbs the high visible photon flux from the laser, which induces photochemical and/or photothermal bond cleavage of side groups and the outgassing of volatile species, predominantly reducing gases such as H_2_, CO, and various hydrocarbons [36]. This process creates a localized pyrolytic microenvironment that is partially shielded from ambient oxygen interference due to the outgassing and facilitates the formation of a three-dimensional, defect-rich, self-standing, and polycrystalline graphene matrix.

Beyond merely facilitating LIG formation, this unique microenvironment also triggers several simultaneous and concomitant chemical and physical interactions between the precursor blend components due to the extremely high temperatures (> 2000 K) and pressures (> 1 GPa) generated during LIG synthesis [22]. While the phenolic resin is pyrolyzed into a conductive LIG matrix, the SiNPs become physically encapsulated within the as-forming LIG structure and exhibit the formation of interfacial covalent bonds. Concurrently, lithium undergoes in situ chemical reactions with the SiNP surface, leading to the formation of a thin prelithiated shell around the embedded SiNPs. The shell remains thin due to the short diffusion time afforded by the rapid laser processing. These interactions will be thoroughly characterized in a later section of this manuscript.

This approach builds upon prior demonstrations of LIG-based composite synthesis, where the unique microenvironment generated during LIG formation has been shown to facilitate various in situ physical and chemical modifications, enabling functionalization of LIG composites for energy storage and conversion. Previous studies have utilized LIG synthesis to trigger various processes, such as the in situ sublimation and covalent integration of molecular adducts [26, 27] (e.g., phosphorus and sulfur) in high amounts into the forming LIG matrix, as well as the physical encapsulation of pre-synthesized nanoparticles [24, 25]. Furthermore, these conditions can enable the direct reduction, nucleation, and encapsulation of metal cation precursors, leading to the controlled formation of reduced metal nanoparticles within the LIG framework [28]. Collectively, these processes result in the synthesis of monolithic, self-standing composites with highly intimate physical or chemical bonds, enhancing structural integrity and electrochemical performance. These reported mechanisms now serve as the foundation for the laser-triggered simultaneous prelithiation and encapsulation strategy demonstrated in this work.

Moreover, Fig. 1c showcases a large-format (20 cm length) Cu foil coated with the precursor slurry and the resulting laser-processed product, highlighting the scalability of the approach. This demonstration underscores the potential for seamless integration of the prelithiated composite synthesis into existing roll-to-roll manufacturing lines, paving the way for high-throughput, industrial-scale production. Supporting this potential, our current laboratory parameters utilizing a large-format laser bed (80 cm × 50 cm) already allow for fabrication rates exceeding hundreds of cm^2^ per hour. Beyond fabrication speed, the process offers superior material utilization compared to conventional techniques. While the carbonization involves an intrinsic mass loss of ~ 38% from the precursor to the final composite film due to volatile release and lithium salt decomposition, this step effectively concentrates the active silicon. Due to the high thermal stability of silicon relative to the volatile organic byproducts, the active material is not removed but rather retained within the final architecture. This contrasts favorably with chemical vapor deposition (CVD) methods that suffer from low conversion efficiency of gaseous precursors or solution-based synthesis techniques that frequently incur active material losses during filtration or purification. Consequently, our additive-free method ensures 100% utilization of the synthesized active material without post-processing waste.

### Structural and Morphological Analysis

**Fig. 2:**
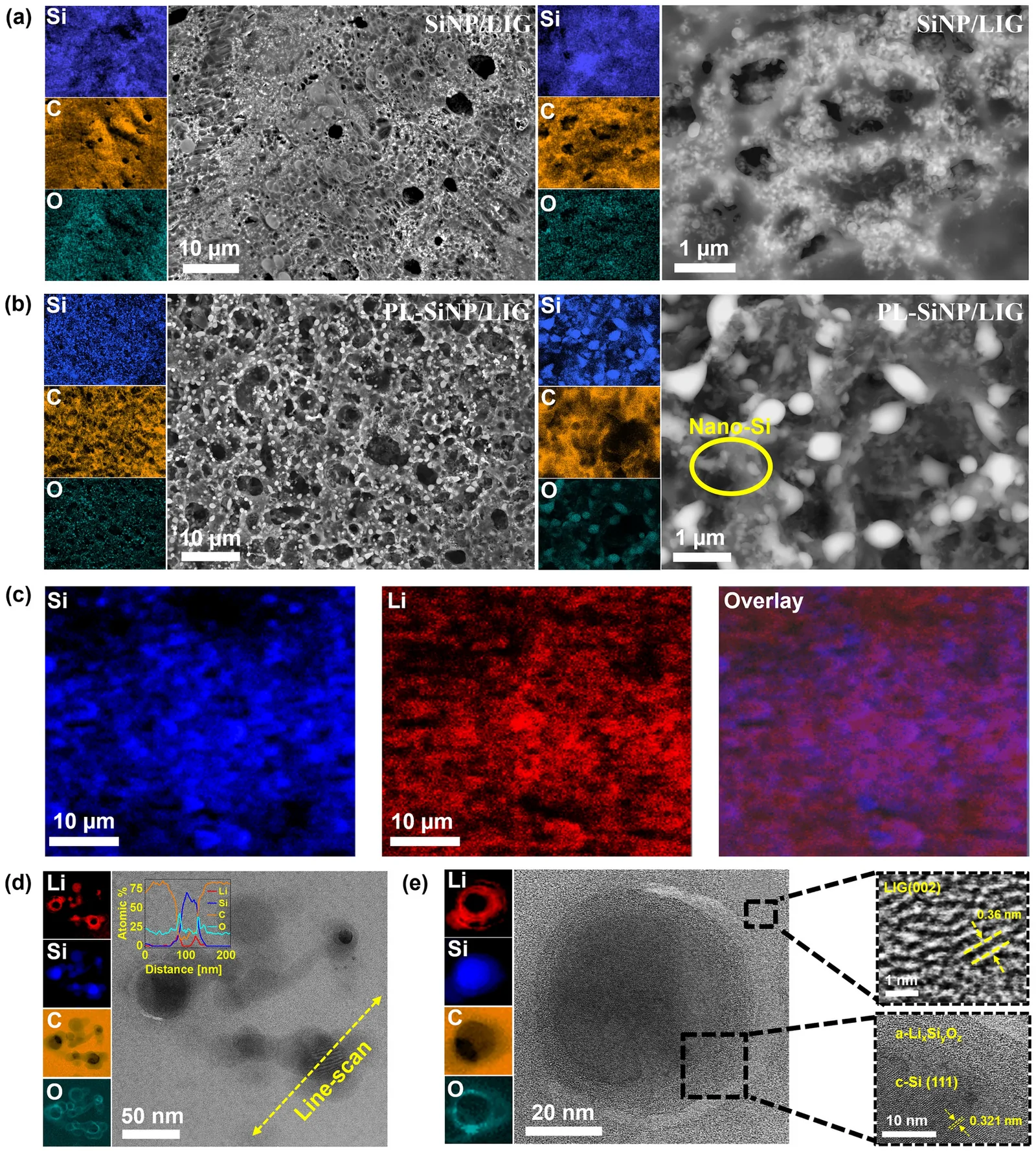
**a** HR-SEM images of the non-prelithiated SiNP/LIG composite at varying magnifications with corresponding EDS maps, highlighting the high porosity and uniform SiNP distribution within the LIG matrix. **b** HR-SEM images of the in situ prelithiated (PL) SiNP/LIG composite with corresponding EDS maps. **c** TOF-SIMS mapping of the PL-SiNP/LIG composite surface at the microscale. **d** Low-magnification STEM images of prelithiated SiNPs within the LIG matrix, with EELS mapping revealing a lithiated SiNP shell. The top-left inset shows a line-scan EELS profile of elemental distribution across the particle diameter. **e** High-magnification STEM image of a prelithiated SiNP with EELS mapping. The top inset shows HR-STEM imaging of the LIG matrix with measured interplanar spacing, and the bottom inset shows the amorphous lithiated shell interface with crystalline Si

The structural and morphological characteristics of both non-prelithiated and prelithiated (PL) SiNP/LIG composites after laser irradiation were investigated using high-resolution scanning electron microscopy (HR-SEM), as shown in Fig. 2a, b, respectively. The corresponding energy-dispersive X-ray spectroscopy (EDS) elemental mapping is also displayed. All images presented in this figure were synthesized using a 1:1 Si:LiOH mass ratio in the precursor blend (for the prelithiated case).

The average diameter of the pristine crystalline SiNPs is approximately 100 nm (shown in Fig. S1). EDS quantification reveals that silicon accounts for more than 35 wt% relative to the total carbon/silicon content. Additionally, cross-sectional analysis shows that the tested electrode thickness increases from approximately 44 µm before laser irradiation to around 55 µm after processing, as shown in Fig. S2. This increase can be attributed to the graphitization of the carbon matrix during laser treatment.

Backscattered electron imaging was employed to emphasize elemental contrast, highlighting the SiNPs (appearing bright) against the darker LIG matrix. The images reveal a highly porous structure in both the non-prelithiated and prelithiated samples, which results from the significant outgassing occurring during the laser processing. This porosity is accompanied by a dense distribution of SiNPs embedded within the conductive LIG matrix. In the prelithiated sample, while the original ~ 100 nm Si nanoparticles remain clearly visible and well-dispersed throughout the structure, additional larger particles, confirmed by EDS to contain silicon, are also observed. Their presence suggests that lithium introduction and the extreme thermal conditions of laser processing may have induced partial particle coalescence and reactivity, leading to the formation of these larger Si-containing domains.

Notably, Li is absent in the EDS mapping because Li has a low atomic number and does not emit a strong characteristic X-ray signal upon electron beam interaction. Accordingly, to accurately determine the homogeneity of the Li and Si distribution within the LIG matrix at the micro-scale, time-of-flight secondary ion mass spectroscopy (TOF-SIMS) was performed on the sample, as shown in Fig. 2c. This, together with the low-magnification HRSEM and EDS mapping results, reveals a homogeneous distribution of Li on the evenly dispersed SiNPs, indicating that the prelithiation process was highly uniform and that Li is well integrated throughout the composite.

This uniform spatial correlation between Li and Si indicates that lithiation occurs homogenously throughout the composite network rather than being localized. In the prelithiated sample, the appearance of a few larger Si-containing particles may result not only from thermal coalescence but also from a reaction between Si and Li during laser processing. Such reactions, occurring under extreme temperatures, could promote local melting and volume expansion, driving partial particle coalescence. This growth may result from rapid melting at the ultra-high temperatures of LIG formation (> 2000 K) [23], followed by coalescence and solidification, facilitating atomic diffusion and forming irregular structures compared to the initial spherical SiNPs.

To further elucidate the nanoscale structure and confirm the Li distribution within the composite, scanning transmission electron microscopy (STEM) imaging was performed, as presented in Fig. 2d, e. Given that EDS is insufficient for Li detection, electron energy loss spectroscopy (EELS) mapping was employed to accurately map and quantify Li distribution.

The samples for STEM analysis were extracted and thinned using a focused-ion beam (FIB) to enable both high-resolution imaging and accurate elemental quantification. In Fig. 2d, the displayed EELS line scan quantification of a lithiated SiNP was specifically sectioned using FIB to expose its core, allowing for a cross-sectional view of the lithiated nanoparticle and silicon core. This preparation method enables accurate assessment of the lithiation extent and elemental distribution within the nanoparticle.

The EELS mapping reveals Li presence in the composite, with a particularly strong signal on the outer shell of the SiNPs. This further suggests partial lithiation of the SiNPs, likely driven by the extreme temperatures and reactive environment generated during lasing, but limited by the ultra-fast kinetics of the laser process. The EELS line-scan quantification analysis demonstrates a distinct increase in Li and Si signal intensity as the scan approaches the nanoparticle surface from the surrounding LIG matrix. This indicates the presence of a boundary region, approximately 10 nm in thickness, where lithiation has occurred.

Figure 2e displays the lithiated SiNP, where Li, Si, and O EELS signals overlap, suggesting the formation of a Li_x_Si_y_O_z_ core–shell structure. The core of the nanoparticle remains largely elemental Si, while the outer shell incorporates Li and O. Quantitative analysis of this shell region indicates a Li content of approximately 13 at%, confirming partial lithiation.

To further characterize the LIG matrix, high-resolution STEM (HR-STEM) analysis was conducted. As shown in the top inset of Fig. 2e, the measured interplanar distance within the (002) plane of the LIG is approximately 0.36 nm, which is slightly enlarged compared to pristine LIG formation [37].

Finally, the bottom inset of Fig. 2e provides HR-STEM imaging of the interface between the depicted crystalline Si core and the surrounding amorphous Li_x_Si_y_O_z_ shell. This structural contrast displays the core–shell nature of the lithiated SiNPs, with the interface exhibiting a clear transition from the pristine crystalline silicon lattice to the amorphous lithiated phase. The presence of this distinct interface confirms that the laser irradiation process facilitated an in situ lithiation reaction, resulting in a structurally integrated core–shell morphology within the composite, all while simultaneously being encapsulated by the as-forming LIG matrix.

Notably, the core of the SiNP appears to have remained unaffected during the lasing process, showing no signs of amorphization or oxidation. This is significant, as it suggests that the core retains its original crystalline structure, which is crucial for maintaining the high lithiation capability of pristine Si. This observation aligns with our previous works depicting the identical process performed without Li salt addition to the blend [24, 25], further supporting the robustness of this approach in preserving the core structure of the SiNPs.

These structural insights collectively demonstrate that the low-energy laser-induced processing of the ternary blend successfully yields a highly porous, conductive LIG matrix with uniformly distributed and partially lithiated SiNPs, forming a composite material with potential advantages for electrochemical applications.

Additional structural and spectroscopic analyses were performed on both non-prelithiated and prelithiated SiNP/LIG composites to further investigate their material properties and highlight key differences. All analyses presented here were conducted on samples synthesized using a 1:1 Si:LiOH mass ratio in the precursor blend.

**Fig. 3:**
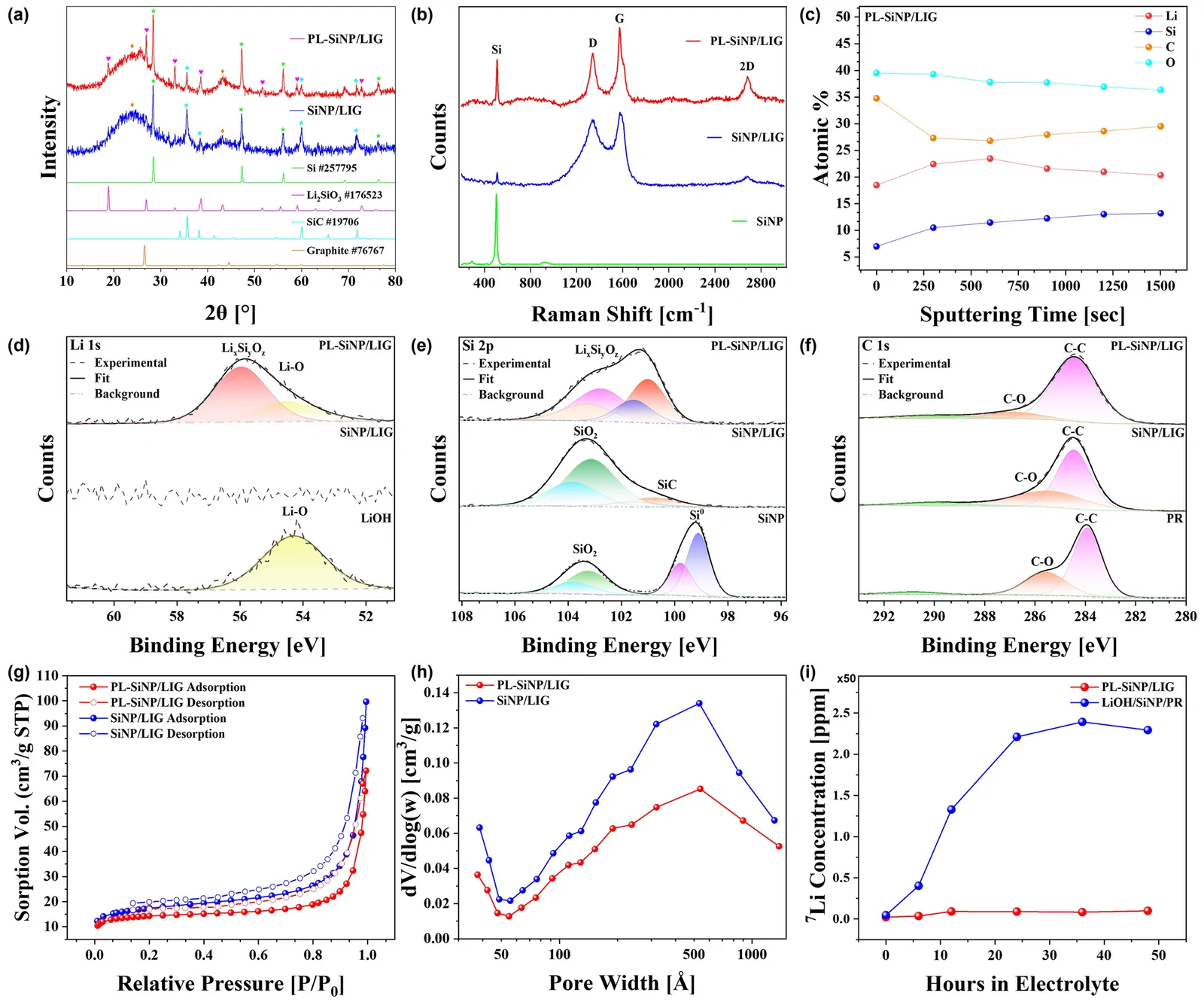
**a** PXRD patterns of PL-SiNP/LIG and SiNP/LIG with ICSD reference peaks. **b** Raman spectra of PL-SiNP/LIG, SiNP/LIG, and pristine SiNPs. **c** XPS depth profile showing atomic percentages of Li, Si, C, and O in PL-SiNP/LIG. **d** XPS Li 1*s* spectra of PL-SiNP/LIG, SiNP/LIG, and LiOH precursor. **e** XPS Si 2*p* spectra of PL-SiNP/LIG, SiNP/LIG, and SiNP precursor. **f** XPS C 1*s* spectra of PL-SiNP/LIG, SiNP/LIG, and phenolic resin (PR) reference. **g** BET N_2_ adsorption–desorption isotherms of PL-SiNP/LIG and SiNP/LIG. **h** BJH pore size distribution of PL-SiNP/LIG and SiNP/LIG. **i** ICP-MS analysis of Li^+^ concentration in electrolyte over time for PL-SiNP/LIG and the unirradiated LiOH/SiNP/phenolic resin precursor blend after immersion

Figure 3a displays the powder X-ray diffraction (PXRD) patterns of the SiNP/LIG and PL-SiNP/LIG composites, which were examined to identify the primary bulk crystalline phases and assess any impact on the crystalline Si active material used in the anode. All peaks were identified using the Inorganic Crystal Structure Database (ICSD), with reference numbers provided accordingly in the graph.

In both composites, the primary (002) peak of the LIG structure appears at a 2*θ* value of approximately 24.6°, corresponding to an interlayer graphene spacing of ~ 0.36 nm, as determined using Bragg’s law (Eq. 1 in the methodology section). The broad nature of the peaks suggests a highly polycrystalline graphene structure, with the full-width at half-maximum (FWHM) used to estimate the average crystallite size via Scherrer’s equation (Eq. 2 in the methodology section). The crystallite size in the prismatic direction (*L*_*c*_), calculated from the (002) peak, was approximately 0.79 nm, indicating an average of approximately two graphene layers. In the basal plane, the domain size (*L*_*a*_), determined from the (100) peak at 43.5°, was approximately 14 nm, confirming the nanocrystalline nature of the LIG.

The PXRD patterns of both composites primarily display characteristic peaks of elemental Si, confirming that the bulk of the SiNPs remained largely unaffected by the lasing process and continue to be the dominant crystalline component. Notably, the absence of any SiO_2_ peaks indicates that significant oxidation did not occur during laser treatment, a result attributed to the reducing carbothermal atmosphere generated during LIG synthesis, as shown in previous studies [28].

In addition to elemental silicon, several smaller but distinct peaks consistent with crystalline silicon carbide (SiC) are observed, formed due to the extreme temperatures and pressures generated during LIG formation. The presence of SiC further demonstrates the robustness of the process in creating a pseudo-monolithic structure characterized by a strong physical and covalent interface between the composite components. This observation suggests that the laser-triggered process facilitates high-temperature reactions typically achieved only through conventional methods, which often involve mixing silica and petroleum coke followed by heating in furnaces at temperatures up to 2500 °C [38].

However, the key difference between the prelithiated and non-prelithiated samples is the emergence of prominent peaks of crystalline lithium metasilicate (Li_2_SiO_3_), alongside the amorphous lithium silicate phases observed in the STEM-EELS analysis. This further confirms that an in situ chemical reaction has occurred between the Li salt and the silicon surface during the LIG formation. Furthermore, no crystalline LiOH peaks are present, indicating that the reaction proceeded to completion, effectively incorporating Li into the composite structure without leaving residual unreacted LiOH.

Raman spectroscopy of SiNP/LIG and PL-SiNP/LIG composites, shown in Fig. 3b, was conducted to assess the quality and structural characteristics of the graphene. The G band (~ 1580 cm^−1^) corresponds to the in-plane vibrational mode of *sp*^2^-hybridized carbon atoms, indicating graphitic crystallinity, with higher intensity correlating to greater electrical conductivity. The D band (~ 1350 cm^−1^), associated with out-of-plane vibrational modes, reflects defects such as vacancies, grain boundaries, *sp*^3^-hybridized carbon sites, and doping-induced irregularities. The 2D band (~ 2700 cm^−1^), an overtone of the D band, provides insights into graphene layer stacking and overall material quality [39].

LIG-based materials typically exhibit a prominent D band due to the ultrafast kinetics of laser-induced pyrolysis [21]. The rapid heat dissipation in this process limits nucleation and diffusion, leading to the formation of nanometric polycrystalline domains of multilayer graphene, as confirmed by STEM and PXRD analyses. These domains contain numerous defect sites and grain boundaries, forming a three-dimensional structure with differential orientations. The intensity ratio of the D and G bands (I_D_/I_G_) is 0.65 for PL-SiNP/LIG and 0.83 for SiNP/LIG, suggesting a slightly higher degree of graphitization in the prelithiated sample. The improved graphitization (lower I_D_/I_G_ ratio) in the prelithiated samples likely stems from the LiOH precursor. The alkaline environment promotes a denser, highly crosslinked polymer network rich in methylene bridges [40]. This robust structure provides a noticeable advantage during pyrolysis. Literature indicates that such crosslinking reinforces the carbon backbone and has been shown both theoretically and experimentally to yield superior graphitization [41–43]. This pre-ordering of the polymer backbone is particularly relevant in the context of our ultrafast laser pyrolysis. Unlike conventional slow furnace carbonization, where molecular chains have time to relax and reorganize, the laser process imposes a rapid thermal shock. A highly crosslinked network may allow the carbon backbone to fuse into additional ordered graphene domains within milliseconds rather than fracturing into amorphous defect clusters.

While bulk composition analysis is crucial for battery applications, given that battery reactions are predominantly bulk rather than surface-controlled, a thorough analysis of surface composition is also vital. Understanding the impact of prelithiation on surface composition and possible artificial SEI formation is critical for numerous battery performance aspects, including charge transfer kinetics, barriers to Li-ion diffusion, and SEI formation stability and integrity. Consequently, X-ray photoelectron spectroscopy (XPS) was conducted to accurately characterize these surface-related phenomena at the nanoscale.

First, delicate Ar^+^ ion cluster sputtering was conducted, providing depth analysis as shown in Fig. 3c, spanning tens of nanometers from the surface. This analysis yielded average atomic percentages of 11.5%, 21.1%, and 37.6% for Si, Li, and O, respectively, indicating the presence of lithium silicate phases and possibly other Li-containing compounds on the surface. The Li 1*s* spectrum in Fig. 3d shows a significant shift from the LiOH precursor peak at ~ 54.2 eV to another major peak emerging at ~ 55.8 eV, which several previous studies have attributed to lithium silicate phases [44–46].

The Si 2*p* spectrum in Fig. 3e illustrates the surface composition of pristine SiNPs, SiNP/LIG, and PL-SiNP/LIG. While the bulk of the Si material in both composites, as confirmed by PXRD and Raman spectroscopy, remains elemental Si, the surface has clearly transformed chemically and now exhibits higher binding energy phases consistent with higher oxidation states. The non-prelithiated sample displays peaks corresponding to SiO_2_ and SiC at approximately ~ 103.2 and ~ 101 eV, respectively, consistent with the measured pristine SiNPs and previous literature reports [47].

The prelithiated sample, however, exhibits a significantly stronger peak at around ~ 101 and ~ 102.8 eV, further confirming the formation of various lithium silicate phases [19, 45, 46] alongside silicon carbide. Due to the inherently unpredictable and ultra-fast laser-triggered prelithiation process, multiple amorphous lithium silicate compositions (Li_x_Si_y_O_z_), as observed in EELS analysis, may coexist alongside crystalline phases, with the overall peak ranges aligning with findings from prior literature.

In the C 1*s* spectrum shown in Fig. 3f, the phenolic resin (PR) LIG precursor, SiNP/LIG, and PL-SiNP/LIG samples were compared to analyze the chemical properties of the LIG. Both non-prelithiated and prelithiated samples predominantly exhibit *sp*^2^-hybridized carbon, indicated by the main peak at ~ 284.4 eV, and show clear evidence of pyrolysis during laser processing, as demonstrated by the significant reduction of *sp*^3^-hybridized C-O and O=C–O bonds relative to the resin precursor. These results, in conjunction with PXRD and Raman analyses, indicate that the prelithiation process did not negatively impact the quality or structural integrity of the LIG.

Gas adsorption and desorption isotherms obtained using nitrogen (N_2_) and analyzed via the Brunauer–Emmett–Teller (BET) method (Fig. 3g**)** were conducted to evaluate the specific surface area (SSA) and pore size distribution of SiNP/LIG and PL-SiNP/LIG composites. In Si-based anodes, SSA plays a crucial role, as higher values enhance electrolyte infiltration and reaction kinetics, while excessive SSA can lead to increased electrolyte decomposition and SEI formation. A moderate SSA is generally desirable to balance these factors and support stable electrochemical performance.

BET analysis reveals that both SiNP/LIG and PL-SiNP/LIG composites exhibit similar SSA values of 62.8 and 53.3 m^2^ g^−1^, respectively. This falls within the moderate range for lithium-ion battery applications and compares well with conventional silicon-carbon composites, where excessively high SSA can lead to increased electrolyte consumption [48, 49].

The Barrett–Joyner–Halenda (BJH) pore size distribution, extrapolated from the isotherms in Fig. 3h, provides further insights into the material’s porosity. A well-developed porous structure enhances ion transport and accommodates the ~ 400% volume expansion of silicon, mitigating pulverization and preserving electrode integrity during cycling. Both samples exhibit mesoporous structures. The SiNP/LIG composite shows a higher total pore volume compared to the PL-SiNP/LIG composite, suggesting that lithiated species formed during laser processing may occupy part of the available pore space. Both samples display characteristic Type IV isotherms with H3-type hysteresis loops, indicative of slit-like mesopores. The slight reduction in mesopore volume in the prelithiated sample supports this interpretation and suggests structural modifications resulting from the reaction with lithium.

Figure 3i shows ICP-MS analysis conducted to assess changes to Li solubility in the carbonate-based electrolyte of LIBs due to the chemical transformation of Li species after the lasing process. Minimizing lithium solubility is crucial for ensuring the stability of the prelithiated anode, maintaining structural integrity, achieving long-term capacity retention, and enhancing electrochemical performance. ICP-MS, with its high sensitivity and elemental detection capabilities down to the ppb level, is particularly well-suited for accurately detecting solvated Li species, which are often difficult to quantify through other analytical methods.

To accurately evaluate Li dissolution, an experiment was conducted on both a control sample, the precursor ternary blend film before lasing (consisting of PR, SiNPs, and LiOH), and the PL-SiNP/LIG anode after the lasing process. Each sample was immersed in an enclosed vessel containing 20 mL of a standard carbonate-based electrolyte for LIBs. A small amount of electrolyte was periodically extracted from each vessel over the course of multiple days for analysis.

ICP-MS measurements of the extracted electrolyte, specifically monitoring the presence of ^7^Li (diluted by a factor of 50), revealed no discernible increase in Li concentration from the sample after laser irradiation, further confirming the insolubility of the transformed Li chemical species. In contrast, the non-irradiated control sample exhibited a steady increase in Li concentration over time, indicating LiOH dissolution into the electrolyte.

### Effect of Si:Li Ratios on Anode Performance

**Fig. 4:**
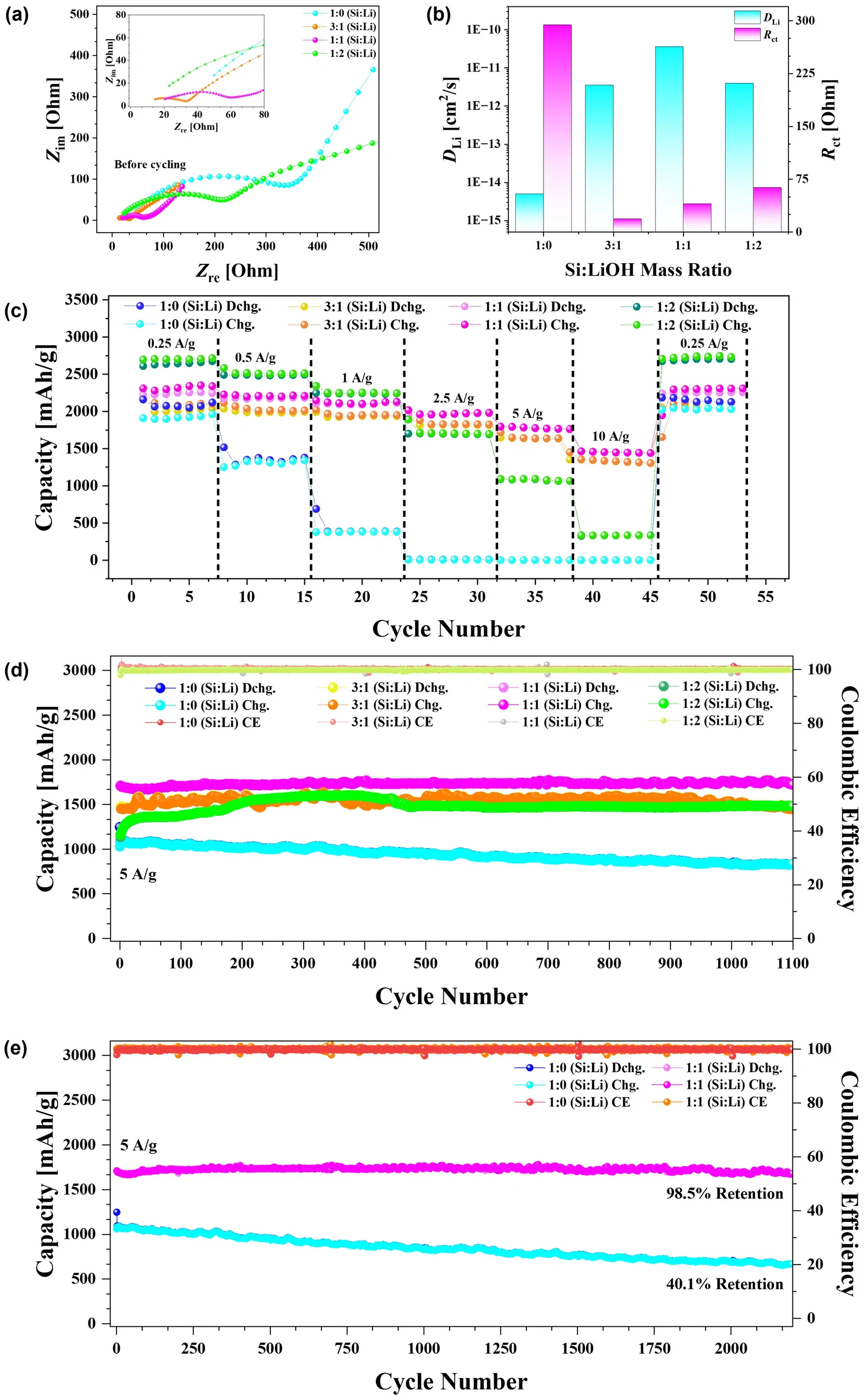
**a** PEIS analysis of PL-SiNP/LIG composites at varying Si:LiOH mass ratios before cycling, with the equivalent circuit model shown in Fig. S3. **b** Li-ion diffusion coefficients (*D*_Li_) and charge transfer resistance (*R*_ct_) for PL-SiNP/LIG anodes using different Si:LiOH ratios, extracted from the EIS plots. **c** Rate performance of PL-SiNP/LIG anodes using different Si:LiOH ratios. **d** Galvanostatic cycling of PL-SiNP/LIG anodes using different Si:LiOH ratios at 5 A g^−1^ for 1100 cycles. **e** Continuation of the galvanostatic cycling conducted in **d** for the 1:1 and 1:0 samples for 2000 + cycles

The electrochemical performance of PL-SiNP/LIG anodes in half-cells was evaluated extensively using different SiNP:LiOH mass ratios in the initial precursor slurry to assess the impact on ion transport, charge transfer kinetics, and overall electrochemical stability, as shown in Fig. 4. All electrochemical analyses presented in this section were conducted using LiOH as the Li salt precursor. This choice was made to establish a baseline understanding of the impact of Li salt addition at different ratios on the electrochemical performance of PL-SiNP/LIG anodes. Later in the manuscript, we will explore the effect of different Li salt precursors, evaluating how variations in precursor chemistry influence key electrochemical properties.

Figure 4a displays potentiostatic electrochemical impedance spectroscopy (PEIS) analysis of the anodes, with the equivalent circuit model presented in Fig. S3. These measurements were performed before cycling and after a sufficient rest period. To accurately assess activity within the Faradaic region, measurements were conducted at ~ 0.2 V vs. Li/Li⁺. Figure S4 highlights the linear relation of the impedance to the Nyquist plot’s low-frequency region (~ 0.1–1 Hz), in accordance with Eq. 3 from the Experimental section.

From this linear slope, the Warburg constant, representing semi-infinite Li-ion diffusion within the system, was extracted. A smaller Warburg constant corresponds to improved diffusion, indicating enhanced ion transport. Particularly, the composite prepared without any LiOH addition exhibits a drastically higher Warburg constant compared to all Li-containing samples, indicating significantly hindered diffusion behavior due to the absence of Li-induced structural and electrochemical modifications.

Figure 4b summarizes the findings from Figs. 4a and S4, presenting the Li-ion diffusion coefficients (*D*_Li_) extracted from the Warburg constant using Eq. 4 in the Experimental section, alongside charge transfer resistance (*R*_ct_) values obtained from the EIS fitting. The latter is primarily represented by the diameter of the depressed semicircle in the high-frequency region of the Nyquist plot, reflecting the ease of charge transfer at the electrode–electrolyte interface. As this analysis was conducted prior to cycling, a second semicircle at high frequencies, typically associated with SEI formation, should not be present. However, the impedance response may include an artificial SEI contribution merged within the larger semicircle.

Notably, differences between SiNP:LiOH ratios emerge, where the lowest *R*_ct_ is observed for the 3:1 Si:LiOH ratio, reaching approximately 19 Ω, suggesting optimal charge transfer characteristics. However, the highest *D*_Li_ is exhibited by the 1:1 ratio, with a value of 3.6 × 10^−11^ cm^2^ s^−1^, demonstrating superior Li-ion diffusivity while still maintaining a relatively low *R*_ct_ of approximately 42 Ω. In contrast, the 1:0 Si:LiOH composition shows a significantly lower *D*_Li_ of 4.5 × 10^−15^ cm^2^ s^−1^, indicating severely hindered Li-ion transport due to the absence of Li-induced structural modifications. This trend suggests a trade-off between charge transfer resistance and Li-ion diffusivity, where the 1:1 ratio represents an optimized balance between these two competing factors. Moreover, the diffusivity observed (via EIS) in the 1:1 composition surpasses that of previously reported prelithiated silicon/carbon anodes by at least an order of magnitude [50, 51].

Importantly, the effect of lithium silicate species on the surface of the SiNPs, the key compositional distinction between the prelithiated and non-prelithiated anodes, on battery performance warrants particular attention. Such species, often formed through diverse routes such as thermal lithiation, chemical prelithiation, or electrolyte decomposition in previous studies, have been widely shown to play a beneficial role in stabilizing silicon–electrolyte interfaces by acting as ion-permeable yet chemically robust interphases that suppress continuous electrolyte decomposition and mitigate capacity fade [52–54]. These lithium silicate layers also accommodate volume expansion and serve as mechanically compliant buffers that relieve interfacial stress during repeated lithiation–delithiation cycles, maintaining electrical contact and suppressing fracture.

Li_2_SiO_3_ and related Li_x_SiO_y_ species are also known to form naturally within the inner SEI, near the Si surface, of conventional Si anodes, where they provide chemical and mechanical stabilization of the electrode–electrolyte interface [55–57]. As reported by Cao et al. [57], a smooth LiₓSiOᵧ layer as the bottom SEI can promote the growth of a conformal, thin, and uniform outer SEI, thereby improving both cycling stability and rate capability. Thus, the preformation of Li_2_SiO_3_ in the prelithiated SiNP/LIG anode ensures that this beneficial component is already integrated within the inner SEI prior to cycling, reducing the extent of electrolyte-driven lithiation and minimizing initial lithium consumption during SEI formation. This preserves more active lithium for reversible storage and contributes to higher initial coulombic efficiency.

These findings highlight the critical role of Li salt addition and ratio in modulating the electrochemical properties of the composite, with specific ratios enhancing either ion transport or charge transfer kinetics based on the interplay of structural and electronic effects.

High ionic conductivity, as characterized by the diffusion coefficient, ensures rapid Li-ion mobility through the electrode structure, while high electronic conductivity, evaluated through charge transfer resistance, facilitates efficient electron flow at the electrode–electrolyte interface. The interplay between these properties ultimately determines the anode’s ability to sustain performance at increasing current densities without significant polarization or capacity loss.

Accordingly, Fig. 4c presents the rate performance analysis of PL-SiNP/LIG anodes at varying Si:LiOH mass ratios, conducted to assess the influence of prelithiated SiNPs and Li reservoirs on charge–discharge behavior under varying current densities. Rate performance is a crucial parameter as it directly reflects the electrode’s ability to sustain high-power operation while maintaining efficient Li-ion transport and charge transfer.

A clear distinction is observed between the anodes, with the 1:0 Si:LiOH ratio displaying vastly inferior rate performance compared to the lithiated ratios. This sample not only exhibits lower overall gravimetric capacity even at low current densities but also shows pronounced capacity drops with increasing current, ultimately demonstrating complete capacity loss beyond 2.5 A g^−1^. This suggests that mass transport limitations and sluggish charge transfer kinetics severely restrict its ability to sustain performance under high-power conditions.

In contrast, all Li-containing samples display improved rate capability, further confirming the positive impact of Li addition on electrode kinetics. The 1:2 Si:Li ratio demonstrates relatively high capacity at low current densities but exhibits a sharp decline starting from 5 A g^−1^, with significant capacity loss at 10 A g^−1^. This aligns with the fact that this ratio exhibited the highest *R*_ct_ and lowest *D*_Li_, indicating that while higher Li content might enhance initial capacity, excessive Li incorporation can lead to slightly decreased electronic and ionic transport, ultimately hindering high-rate performance.

Among the varying ratios, the 1:1 Si:Li ratio exhibits slightly superior rate performance compared to the 3:1 ratio, likely attributed to its significantly higher *D*_Li_, which facilitates more efficient Li-ion transport despite having a marginally higher *R*_ct_. This balance between diffusion and charge transfer enables sustained capacity retention even at elevated current densities, highlighting the importance of optimizing both parameters. Notably, the 1:1 composition demonstrates unprecedented capacity retention across a wide range of rates, retaining approximately 63% of its capacity when increasing the current density from 0.25 to 10 A g^−1^, underscoring its exceptional performance from slow to ultra-fast charging regimes. Overall, the differences between specific Si:LiOH ratios suggest that while any level of Li addition is beneficial, optimizing the ratio is crucial for achieving the best balance between capacity retention, charge transfer kinetics, and long-term cycling stability.

Figure 4d presents galvanostatic cycling performance of PL-SiNP/LIG anodes at a high current density of 5 A g^−1^, evaluating ICE, gravimetric capacity, and long-term stability over 1100 cycles for different Si:LiOH ratios in the precursor blend. As shown in Fig. S5, the electrode without Li addition (1:0) delivers the lowest ICE of 83%, whereas introducing Li markedly enhances performance, yielding ICE values of 94%, 97%, and 97% for ratios of 3:1, 1:2, and 1:1, respectively. These results clearly demonstrate that incorporating Li improves the first-cycle efficiency.

This finding is further consistent with previous reports on Li-silicate layers in Si-based anodes for Li-ion batteries, which demonstrated that such interfacial species not only enhance charge-transfer kinetics, Li-ion diffusion, and structural stability, but also serve as a key in situ–formed SEI component [55–57]. By mitigating the additional Li consumption typically required to form a stable SEI, the Li-silicate layer effectively improves the initial Coulombic efficiency while maintaining long-term interfacial stability.

Figure S6 further presents representative voltage profiles at selected cycles for all cells, illustrating the stable and reproducible electrochemical behavior of the prelithiated anodes throughout long-term cycling. This observation further suggests that Li addition helps reduce irreversible Li loss and SEI formation, contributing to better electrode stabilization during early cycling. Furthermore, all prelithiated samples demonstrate an average coulombic efficiency exceeding 99.9%, indicating highly reversible Li-ion reactions and exceptional electrochemical and structural stability during prolonged cycling.

For long-term capacity retention, the 3:1 Si:LiOH ratio exhibits a gravimetric capacity of approximately 1446 mAh g^−1^ after 1100 cycles, while the 1:1 ratio maintains a higher capacity of approximately 1702 mAh g^−1^. The 1:2 ratio follows with a stable capacity of 1484 mAh g^−1^. All compositions display excellent cycling stability with minimal capacity fading (> 98%), with some even showing a slight increase in capacity over time, which is often attributed to gradual activation of the electrode material or improved Li-ion transport pathways. Furthermore, all samples demonstrate an average coulombic efficiency exceeding 99.99%, confirming highly reversible Li-ion reactions and exceptional electrochemical stability and structural durability over extended cycling. In contrast, the 1:0 composition without any LiOH addition exhibits a substantially lower capacity of 802 mAh g^−1^ after 1100 cycles, with only 77% retention. The disparity in performance emphasizes the substantial role of Li addition in improving electrode structure, enhancing Li-ion diffusion, and mitigating capacity degradation over prolonged cycling. Notably, Fig. 4e presents extended cycling data for both the 1:1 and 1:0 compositions over 2000 + cycles. The 1:1 prelithiated anode retains 98.5% of its capacity, while the 1:0 non-prelithiated anode retains only 60.1%. Figure S7 further demonstrates continued cycling of the same cells up to 4150 cycles, where the 1:1 prelithiated sample retains an outstanding 83% capacity, substantially higher than the 40% retention observed for the non-prelithiated counterpart.

The enhanced electrochemical performance of the prelithiated anode is further supported by cyclic voltammetry conducted with and without prelithiation, as shown in Fig. S8. While all lithiation and delithiation peaks appear at the same voltages, the prelithiated sample exhibits a much stronger delithiation peak at ~ 0.34 V and a weaker peak at ~ 0.51 V, whereas the non-prelithiated sample shows the opposite pattern. A previous report identified the ~ 0.34 V peak as corresponding to delithiation from amorphous lithium silicide, and the ~ 0.5 V peak as arising from the dealloying of crystalline lithium silicide [58]. Nulu et al. later found that a dominant ~ 0.5 V peak is associated with sluggish kinetics and higher interfacial resistance [59]. Similarly, Woodard et al. showed that delithiation from the crystalline phase, indicated by more intense high-voltage peaks, can lead to greater mechanical strain and faster capacity degradation, further supporting the stability advantage of suppressing crystalline phase formation via prelithiation.

Overall, these findings strongly indicate that Li salt addition to the precursor blend is beneficial in any ratio, and that the Si:LiOH mass ratio in the precursor blend must be carefully optimized. A well-tuned ratio ensures the presence of sufficient Li reservoirs to enhance diffusion while preventing excessive impedance that could hinder high-rate performance. This underscores the necessity of systematic evaluation and precise control of Li incorporation to maximize both energy and power density in LIB applications.

Furthermore, it is noteworthy that although the crystalline SiC interphase observed in the XRD analysis is electrochemically inert, it confers a critical net-positive benefit to the composite stability. Functioning as a rigid covalent nano-weld, this thin layer chemically anchors the silicon to the graphene matrix. Previous studies by Zhang et al. [60] and Huang et al. [47] have demonstrated that such encapsulation is beneficial for preventing particle isolation and buffering volumetric expansion, while Yu et al. [61] established that SiC interphases further stabilize the anode by inhibiting parasitic reactions. Crucially, the nanometric thickness of this stabilizing film permits effective electron transfer and lithium diffusion without compromising kinetics. This aligns with findings by Tzeng et al. [62], who observed that SiC-encapsulated Si anodes maintain a low and stable *R*_ct_ throughout cycling, in contrast to pristine silicon which exhibited a sharp increase in impedance due to continuous SEI thickening.

The scalability and practical applicability of the prelithiated SiNP/LIG anode were further evaluated under realistic operating conditions. A 1C rate, a standard benchmark for fast-charging capability, was used to assess performance under high-power operation. Notably, by increasing the silicon content in the initial slurry formulation, the overall silicon weight fraction relative to the combined silicon–carbon content can approach 70%, as confirmed by SEM/EDS analysis (Fig. S9), further enhancing the energy density potential of the composite electrode. As shown in Fig. S9, the prelithiated SiNP/LIG anode with an areal mass of 1 mg cm^−2^ demonstrated stable cycling over 50 cycles with no observable degradation, achieving high areal capacity approaching 1 mAh cm^−2^, even at relatively fast charging rates (0.5C). Furthermore, Fig. S10 presents galvanostatic cycling of a full cell comprising a 1 mg cm^−2^ LiFePO_4_ (LFP) cathode and a prelithiated SiNP/LIG anode, cycled at a 1C rate for over 500 cycles. The full cell exhibits exceptional cycling stability and capacity retention with 500 cycles at a 1C rate, maintaining approximately 115 mAh g^−1^ at the 500th cycle with no measurable capacity degradation. These results underscore the long-term durability and scalability of the prelithiated composite for practical lithium-ion battery systems.

### Effect of Lithium Salt Precursor on Anode Performance

**Fig. 5:**
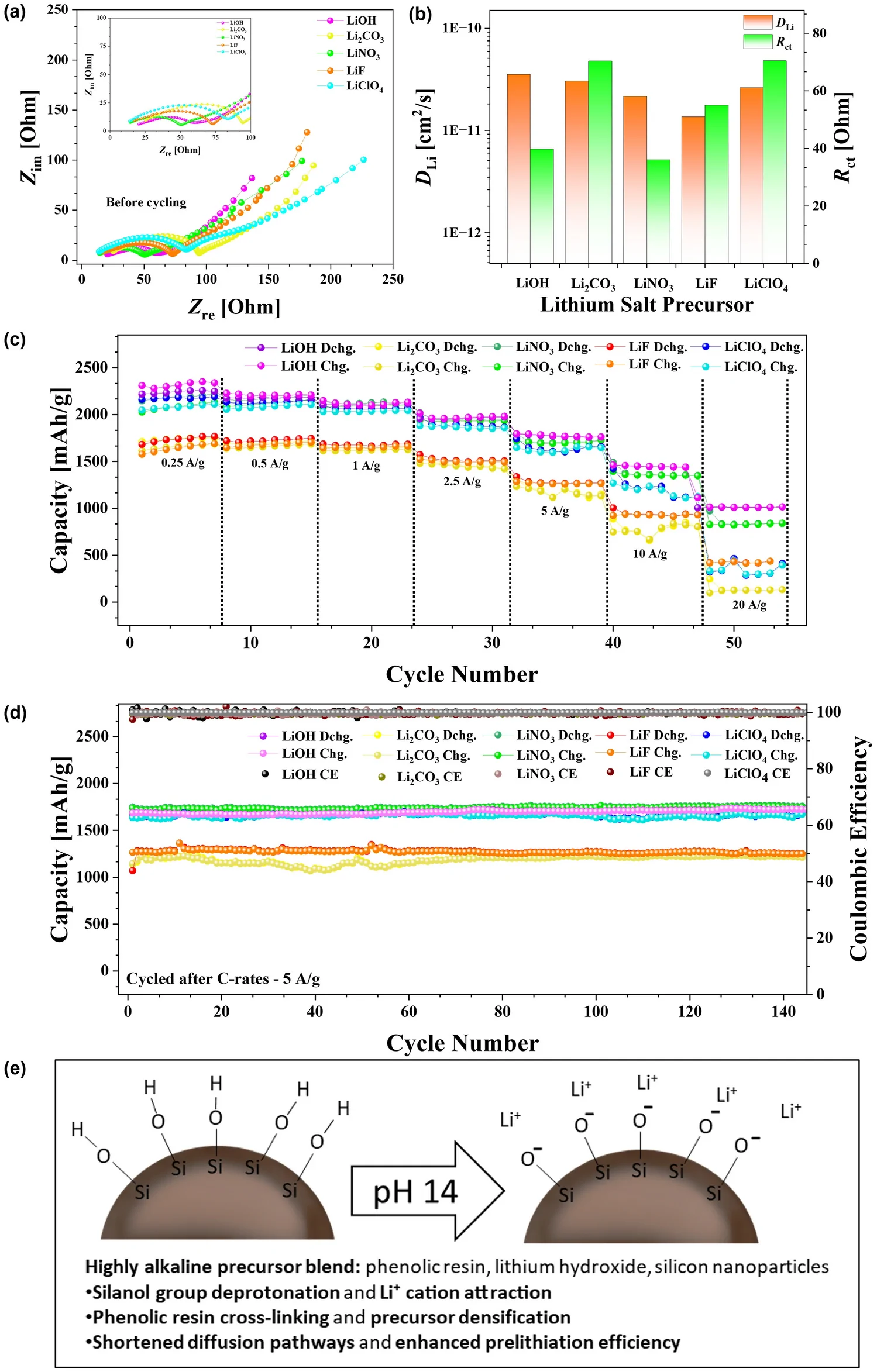
**a** PEIS analysis of PL-SiNP/LIG composites with varying Li precursors before cycling, with the equivalent circuit model shown in Fig. S3. **b** Li-ion diffusion coefficients (*D*_Li_) and charge transfer resistance (*R*_ct_) for PL-SiNP/LIG anodes using different Li precursors. **c** Rate performance of PL-SiNP/LIG anodes using different Li precursors. **d** Galvanostatic cycling of PL-SiNP/LIG anodes using different Li precursors at 5 A g^−1^, cycled after the rate performance analysis. **e** Schematic depicting the role of lithium hydroxide in promoting silanol deprotonation, Li⁺ attraction, resin cross-linking, and enhanced reaction efficiency in the alkaline precursor blend

To further explore the universality of the proposed prelithiated method and its compatibility with common Li salt precursors, it is necessary to assess alternatives beyond LiOH. While LiOH has demonstrated exceptional electrochemical performance, investigating other Li salts provides insight into the broader applicability of this approach. To this end, Li_2_CO_2_, LiNO_3_, LiF, and LiClO_4_ were evaluated as alternative prelithiation sources. Their influence on electrochemical performance in LIBs was systematically analyzed, as shown in Fig. 5.

Compositional and surface analysis was conducted for each prelithiated anode prepared using different lithium salt precursors (Figs. S11–S18), utilizing HRSEM, EDS, XRD, and XPS to provide morphological, structural, and chemical insights into the resulting composites. HRSEM and EDS confirm a uniform dispersion of Si-containing nanoparticles within the LIG matrix. XRD analysis of all samples highlights the broad graphene (002) background together with preserved crystalline Si and SiC formation following laser irradiation. Different lithium species are observed across the various precursor systems, most likely governed by the distinct thermal decomposition behavior and anion volatility of each salt. Li_2_CO_3_ and LiNO_3_ lead to the formation of crystalline Li-silicate phases, whereas LiF remains largely unreacted due to its high thermal stability, and LiClO_4_ results in predominantly amorphous Li-containing species that are not detectable. XPS confirms the surface presence of Li_2_CO_3_ type species near 290 eV in all samples except those derived from LiF, where lithium remains largely in unreacted form. Precursor-dependent heteroatom effects are also observed in the artificial SEI, including C–F bonding for LiF-derived composites, C–N bonding indicating N-doping for LiNO_3_, and residual Cl signatures for LiClO_4_. These results demonstrate that each precursor induces prelithiation through a distinct dominant pathway, either via Li–Si reaction, artificial SEI formation, and/or anion-driven doping, and that, as will be shown in the following electrochemical analysis, the addition of any lithium source clearly improves performance relative to composites prepared without Li salt addition, though the magnitude of enhancement varies depending on the lithium salt chemistry.

Figure 5a presents the PEIS analysis performed before cycling, showing overall similar impedance behavior across all Li salts with slight variations. Figure S19 further examines these differences through the Warburg slope, revealing that all compositions exhibit similar *D*_Li_. Among them, LiOH achieves the lowest slope, corresponding to the highest *D*_Li_, confirming its superior Li-ion transport properties. Figure 5b summarizes both *D*_Li_ and *R*_ct_ extracted from EIS fitting.

Across all Li salts, *D*_Li_ values fall within the range of 3 × 10^–11^–1 × 10^–11^ cm^2^ s^−1^, while *R*_ct_ varies between 35 and 65 Ω. LiOH addition exhibits the highest *D*_Li_, reinforcing its role in promoting efficient Li-ion transport. Meanwhile, LiNO_3_ shows a slightly lower *R*_ct_ than LiOH, suggesting marginally improved charge transfer kinetics. As all these results surpass the performance of the Li-free control shown in Fig. 4, they indicate that while all Li salts contribute positively to electrochemical performance, some offer greater enhancements than others.

Figure 5c further supports the trends observed in diffusion and charge transfer capabilities, showing that LiOH delivers both the highest capacity and the best retention across varying rates, solidifying its superior electrochemical performance. LiNO_3_ follows closely, outperforming the other Li salts in both capacity and retention. Overall, all compositions show significantly improved capacity retention compared to the Li-free control in Fig. 4.

Figure 5d further highlights the universal benefits of Li salt addition, demonstrating excellent cycling stability at a high current density of 5 A g^−1^ over 140 + cycles. In addition to enhancing long-term performance and structural durability, the results confirm improved rate capability, enabling sustained capacity even under fast-charging conditions.

Furthermore, enhanced ICE relative to the non-lithiated samples is observed across all Li-salt systems, as shown in Fig. S20, with ICEs of 92% for the carbonate precursor, 94% for the perchlorate precursor, 92% for the nitrate precursor, and 91% for the fluoride precursor. In addition, all Li-salt-based samples exhibit an average CE exceeding 99.9%, confirming their excellent electrochemical reversibility, stable SEI behavior, and strong structural integrity over extended cycling. While there are some differences in effectiveness among the various salts, the significant improvement observed in each case compared to the no-Li control underscores the versatility and robustness of this method.

Among the tested salts, LiOH consistently exhibits the most favorable electrochemical characteristics. We propose that this arises from its unique chemical ability to modify the precursor environment prior to laser irradiation.

The strongly basic conditions generated by LiOH (conjugate acid H_2_O, pKa ≥ 14) enable effective deprotonation of phenolic hydroxyl groups in the resin (pKa ~ 10) [63] and silanol (Si–OH) groups on the surface of the SiNPs (pKa ~ 4–7) [64], forming negatively charged sites that attract Li⁺ ions and promote interfacial densification within the precursor matrix, as depicted in the schematic displayed in Fig. 5e. Additionally, these alkaline conditions facilitate extensive cross-linking of the phenolic resin through a base-catalyzed addition reaction followed by condensation, resulting in self-crosslinking, a well-documented phenomenon in the literature [40, 65]. This process leads to increased molecular weight and viscosity, ultimately yielding a denser, more compact precursor blend with intimate contact between Li-ions and other blend components. This densification can therefore minimize lithium diffusion distances and enhance interfacial contact during the subsequent ultrafast laser-driven conversion, thereby improving the overall reaction efficiency.

In contrast, the weaker bases Li_2_CO_3_, LiF, LiNO_3_, and LiClO_4_, whose conjugate acids have pKa values of H_2_CO_3_ (pKa_1_ = 6.4, pKa_2_ = 10.3), HF (3.2), HNO_3_ (–1.4), and HClO_4_ (–1.6) [66], lack sufficient alkalinity to induce such deprotonation or ionic association. We propose that their effects on performance therefore arise mainly from secondary phenomena, including variations in thermal decomposition behavior, heteroatom incorporation, or anion volatility during the laser process. As a result, these systems form less compact and more weakly associated precursor networks, which can lead to less uniform Li distribution and less efficient conversion during rapid laser heating.

### Post-mortem Analysis

**Fig. 6:**
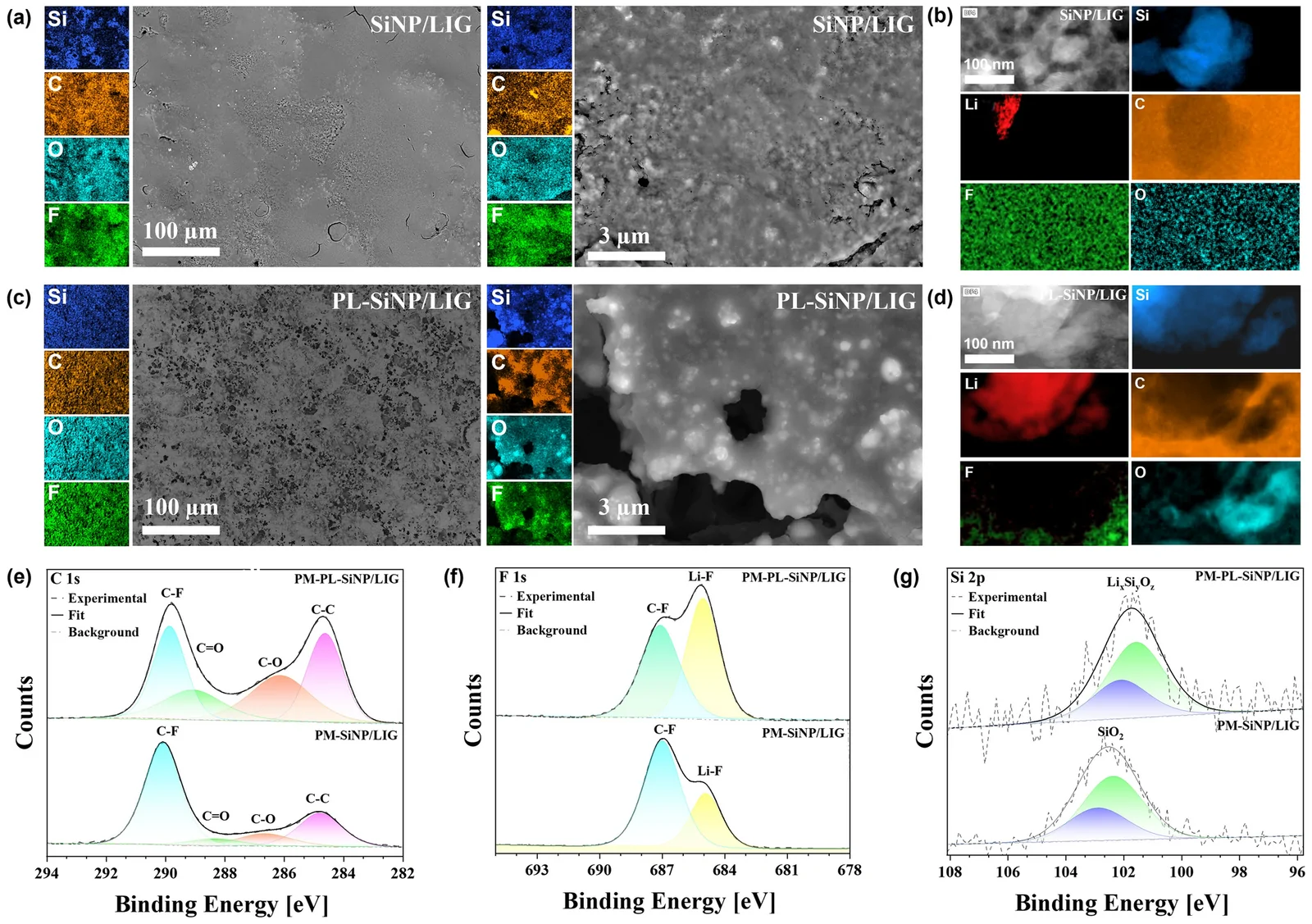
Post-mortem analysis after 100 cycles. **a** SEM/EDS analysis of the post-mortem non-prelithiated anode. **b** STEM/EELS mapping of the post-mortem non-prelithiated anode. **c** SEM/EDS analysis of the post-mortem prelithiated anode. **d** STEM/EELS mapping of the post-mortem prelithiated anode. **e** XPS C 1*s* spectra of both post-mortem prelithiated and non-prelithiated anodes. **f** XPS F 1*s* spectra of both post-mortem anodes after 100 cycles.** g** XPS Si 2*p* spectra of both post-mortem anodes after 100 cycles

Figure 6 provides a comparative post-mortem analysis of the SiNP/LIG and PL-SiNP/LIG anodes after 100 cycles, highlighting key differences in SEI formation and structural integrity. This analysis is critical for evaluating the effectiveness of prelithiation in mitigating the degradation pathways typically associated with silicon-based anodes.

Figure 6a shows SEM images and corresponding EDS maps of the non-prelithiated SiNP/LIG anode. Even at relatively low magnifications, a thick SEI layer is clearly visible, completely covering the originally porous anode structure (see Fig. 2a for the pre-cycling morphology). At higher magnification, the thick SEI coverage becomes even more apparent. The EDS analysis reveals a distinct “backbone” of the previously porous active material, where the Si signal is still detectable. However, this structure is now uniformly overlaid with strong C and F signals, indicating the formation of a dense and continuous SEI.

Minor cracking is also observed, further suggesting that the anode has undergone mechanical stress likely induced by repeated SEI growth, fracture, and reformation, a widely recognized degradation mechanism for silicon-based anodes that leads to continuous lithium consumption, unstable interphases, and rapid capacity fade [67, 68]. Figure 6b presents STEM and EELS mapping of the same non-prelithiated sample to examine lithium distribution. No spatial correlation is observed between the Si and Li signals, and the Li content within the SEI appears to be low.

In contrast, Fig. 6c, d displays the post-mortem analysis of a prelithiated PL-SiNP/LIG anode after 100 cycles, which shows remarkable structural preservation. The porous architecture remains largely intact, closely resembling its pre-cycling state. EDS mapping in Fig. 6c shows that the F signal, corresponding to the various SEI species, conforms to the surface of the anode and does not penetrate or noticeably fill the pore structure. This observation confirms that the SEI in the prelithiated sample is thinner and more uniformly distributed. These results reinforce the conclusion that the laser-triggered prelithiation effectively inhibits a primary degradation mechanism associated with silicon anodes, infinite SEI fracture and reformation, by reducing both the initial lithium consumption and ongoing irreversible lithium loss through the formation of a more stable SEI in the early cycles. Figure 6d further supports this conclusion through STEM/EELS Li mapping of the post-mortem prelithiated anode, which reveals significant spatial overlap between the Li and Si signals. This overlap, resembling the distribution observed in the pre-cycling state, indicates that Li remains closely associated with the silicon framework, suggesting a thinner, more stable interphase and minimal delithiation-induced degradation.

Figure S21 further supports these findings by providing cross-sectional HRSEM and EDS analyses of the PL-SiNP/LIG anodes before and after 50 cycles (delithiated). The post-mortem image shows no discernible increase in electrode thickness, excellent mechanical integrity with no delamination, and minimal SEI growth. The SEI layer remains conformal and uniformly distributed, confirming stable electrode–electrolyte interfaces and validating the superior mechanical stability of the prelithiated system.

XPS was used to further analyze the chemical composition of the SEI on both non-prelithiated and prelithiated anodes. As a highly surface-sensitive technique, XPS is ideal for examining thin interfacial layers like the SEI, providing insight into both elemental composition and chemical states near the electrode surface, typically within the top ~ 10 nm. This makes it especially useful for evaluating SEI thickness, uniformity, and chemical stability.

Figure 6e presents the C 1*s* spectra for both anodes. Two primary peaks are observed: one at ~ 284.4 eV, corresponding to C–C/C=C bonds (mostly from the underlying LIG), and another at ~ 290 eV, attributed to C–F bonds, which are part of fluorinated organic components in the SEI. In the non-prelithiated sample, the C=C peak is significantly suppressed, indicating that the LIG is buried beneath a thick SEI. In contrast, the prelithiated sample shows a much stronger C=C signal, further confirming with this surface-sensitive method that the SEI is thinner and more uniformly distributed, allowing the LIG signal to remain detectable.

Figure 6f shows the F 1*s* spectra. The non-prelithiated sample exhibits a weak LiF peak at ~ 685.5 eV accompanied by a relatively strong peak at ~ 687.5 eV corresponding to C–F bonds in fluorocarbon species within the SEI. In contrast, the prelithiated sample exhibits a more balanced distribution of LiF and organic fluorocarbon signals. Previous studies on silicon anodes have demonstrated that achieving an optimal ratio of inorganic species (such as Li_2_O or LiF) to organic fluorocarbon species in the SEI enhances overall electrochemical performance [69]. Specifically, LiF contributes to the SEI’s mechanical robustness and thermal stability, while the organic components facilitate lithium-ion transport [70]. This chemical balance likely contributes to the improved structural integrity and cycling stability observed in the prelithiated anode.

Figures 6g and S22 show the Si 2*p* and Li 1*s* spectra, respectively. While the peak positions and shapes are broadly similar between the prelithiated and non-prelithiated samples, both before and after cycling, these spectra offer limited additional insight compared to the more informative F 1*s* and C 1*s* spectra.

### Comparative Study

A broader perspective on the effectiveness of this approach can be gained by comparing it with other nano-Si-based prelithiated anodes reported in high-impact publications in recent years. In contrast to other studies that focused on micro-Si or SiO_x_ prelithiation strategies, this section specifically examines nano-Si systems to provide a more relevant benchmark.

A comparative study was conducted to differentiate the proposed laser-triggered in situ prelithiation approach from conventional prelithiation methods for nano-Si-based composites and is displayed in Table 1. The comparison focuses on two key aspects: scalability of the synthesis method and electrochemical performance.

**Table 1:** Comparative study of the performance and synthesis processes of recently reported prelithiated nano-Si–based anodes for LIBs

Original anode	Synthesis method of anode host	Prelithiation method	Li precursor	Anode additives	ICE%	Performance highlights	Year/Reference
SiNP/Laser-induced graphene	Single-step low-energy rapid laser irradiation of SiNP/Phenolic Resin/LiOH blend under ambient atmosphere	Laser-irradiation conducted in situ relative to anode synthesis	Any common Li salts	None	97	1673 mAh g^−1^ @ 5 A g^−1^ with > 98% retention after 2000 + cycles	This work
Si/PTFE/Carbon black blend	SiNPs mixed with binder and conductive additive followed by activation at 200 °C for 2 h then repeated vertical and horizontal rolling into a film	Direct contact prelithiation	Li metal foil	Carbon black and PVDF	100	806.81 mAh g^−1^ @100 mA g^−1^ with capacity retention of 73.79% after 200 cycles	2025/[71]
Commercial SiNP/C powder	Commercial SiNP/C powder mixed with binder and conductive additive	Immersion in lithium-phenanthrene solution	Lithium phenanthrene	Super P and PAA	92.5	270 mAh g^−1^ @1 A g^−1^ with 97.8 retention after 500 cycles	2025/[72]
Si/C NPs	Wet dispersion polymerization method and annealing at 700 °C in N_2_ atmosphere	Direct contact prelithiation	Li metal foil	Super P and PVDF	96.1	1018.4 mAh g^−1^ @0.5 A g^−1^ with 93.5% retention after 500 cycles	2024/[73]
Spherical SiNPs on graphite	Carbonization at 800 °C under inert atmosphere of Siloxane/SiNP composite and then mixed with graphite	"Crushed" metal Li particles were added to anode film and then immersed in localized high concentration electrolyte	Li metal particles	Acetylene Black and styrene butadiene	84	937.5 mAh g^−1^ @ 1 A g^−1^ after 400 cycles, retention not reported specifically but well below 50%	2024/[50]
SiNPs on graphite	Preparation of nickel-laden graphite followed by immersing in LiOH solution and heating and then adding SiNPs and sintering at 1000 °C for 4 h under inert atmosphere	In situ relative to anode synthesis	LiOH	Super P and PVDF	116	~ 600 mAh g^−1^ after 400 cycles, retention and current density not reported	2024/[74]
Carbonized ZIF-8 MOF coated with SiNPs	ZIF-8 MOF synthesis in presence of SiNPs and annealing at 600 °C under Ar atmosphere	Direct contact prelithiation	Li metal foil	PVDF and CNTs	97.9	500 mAh g^−1^ @0.5 A g^−1^ after 100 cycles, retention not stated specifically but under 50%	2024/[75]
Carbonized SiNP graphite/chitosan blend	Mixture of SiNPs, graphite and chitosan carbonized at 800 °C for 1 h in N_2_ atmosphere	Immersion of anode in lithium biphenyl solution at room temp. in glove box	Lithium biphenyl	Super P and PAA	93.9	674.1 mAh g^−1^ @200 mA g^−1^ with 88% retention over 50 cycles	2023/[76]
SiNPs on graphite	CVD of SiNPs with graphite at 550 °C under acetylene flow followed by calcination at 700	Direct contact prelithiation	Li metal foil	Styrene butadiene and Super P	101.5	650 mAh g^−1^ starting capacity @980 mA g^−1^ with 97.1 retention after 200 cycles	2022/[77]
Si/C NPs	Magnesiothermic reduction of silica at 800 °C for 5 min. in air followed by furfuryl alcohol polymerization and carbonization at 600 °C	Direct chemical shorting prelithiation with pressure	Li metal foil	Super C-65 and alginate	97.3	727 mAh g^−1^ @1 A g^−1^ with 90% capacity retention after 500 cycles	2022/[78]
SiNPs wrapped in graphene oxide nanoribbons	Chemical oxidative etching of MWCNTs followed by electrostatic adsorption on SiNPs followed by lyophilization and annealing at 300 °C for 2 h in Ar atmosphere	Direct contact with 1 kg cm^−1^ pressure in Ar atmosphere	Li metal foil	Acetylene black and CMC	97.1	965 mAh g^−1^ @2 A g^−1^ after 500 cycles, retention not reported specifically but at most 50%	2020/[79]
Si/SiO_2_ NPs	High energy mechanical milling under Ar atmosphere	Electrochemical prelithiation through half-cell operation and disassembly	Li metal foil	Acetylene black and PVDF	93.9	992.8 mAh g^−1^ @ 0.5 A g^−1^ with 97% retention after 400 cycles	2020/[80]
SiNP@SiOx core shell	SiNPs oxidized in air at 600 °C for 30 min	Lithium borohydride and SiNP@SiOx placed in autoclave at 750 °C for 10 h	Lithium borohydride	Ketjen black and sodium alginate	89.1	1091 mAh g^−1^ @3 A g^−1^ with 39% retention after 1000 cycles	2019/[46]

From a synthesis and scalability perspective, the proposed method introduces several distinctive advantages. Unlike conventional prelithiation techniques, which typically involve synthesizing a silicon-carbon based composite structure before conducting prelithiation as a separate, ex situ step, this is the first approach to enable composite synthesis and prelithiation simultaneously in a single-step process. During this process, the as-forming LIG encapsulates the SiNPs, while simultaneously facilitating in situ prelithiation. This ensures uniform and intimate integration of Li into the composite structure at the moment of its formation, eliminating the need for additional post-processing steps typically required in chemical or direct-contact prelithiation approaches. As a result, this method streamlines fabrication, enhances reproducibility, and reduces processing complexity.

Another major advantage of this method is that it produces a self-standing, additive-free composite that is directly deposited onto the current collector substrate, making it immediately suitable for battery assembly. This stands in contrast to conventional silicon-based anodes, which rely heavily on binders such as polyvinylidene fluoride (PVDF), carboxymethyl cellulose (CMC), and styrene-butadiene rubber (SBR), along with conductive additives like carbon nanotubes (CNTs) and carbon black derivatives. By eliminating the need for these additives, the proposed approach further simplifies electrode fabrication, reduces material costs, and avoids additional post-processing needs.

Furthermore, the synthesis process is conducted under ambient conditions using rapid, low-energy laser irradiation. Additionally, the universality and simplicity of this technique were demonstrated by showing that any common Li salt can be used to enhance performance. Overall, this approach contrasts sharply with all existing prelithiation techniques, which require either all or at least several of the following: controlled atmospheres, high-temperature or high-pressure treatments, prolonged reaction times, or the use of exotic or hazardous Li sources.

Moreover, as demonstrated in Fig. 1c and previous studies on LIG-based composites, this laser-based technique is well-suited for large-area fabrication through methods such as laser-assisted additive manufacturing or roll-to-roll integration, providing a clear pathway toward scalable and industrially viable battery electrode production.

The key findings from this comparative analysis are summarized in Table 1, which compiles synthesis methods, precursor materials, additive use, and electrochemical performance metrics of various nano-Si-based prelithiated anodes. This comparison highlights the variations in ICE, cycling stability, capacity retention, and rate capability across different strategies.

From a performance perspective, the proposed synthesis approach is not only highly advantageous in terms of simplicity and scalability, but also delivers electrochemical results that clearly exceed those of known nano-Si-based prelithiated anodes reported in recent years. Its long-term cycling stability is unmatched, with minimal capacity fade over thousands of cycles, and its capability at high charge/discharge rates far surpasses that of comparable systems.

Furthermore, the results indicate that complex, energy-intensive synthesis routes and problematic Li precursors are not necessarily required to achieve high-performance prelithiated anodes. This underscores the effectiveness of a streamlined, scalable, and low-energy approach for achieving superior electrochemical performance without the need for excessive processing complexity.

## Conclusions

This study presents the first demonstration of a laser-based, ambient, solid-state, and in situ prelithiation technique for silicon-graphene composite anodes. By leveraging the extreme localized thermal and pressure conditions generated during LIG formation, this single-step approach simultaneously achieves porous graphene matrix synthesis, SiNP encapsulation, and prelithiation under atmospheric conditions, without the need for binders, conductive additives, hazardous lithium sources, or controlled environments. The resulting prelithiated anodes exhibit outstanding electrochemical properties, including high ICE, enhanced Li-ion diffusion, and near-zero capacity decay over 2000 + cycles at high current densities, surpassing the performance of non-prelithiated analogs and other reported nano-Si anodes.

Structural and spectroscopic analyses confirm the formation of a self-standing, additive-free PL-SiNP/LIG composite comprising partially lithiated SiNPs embedded within a conductive, porous LIG matrix. EELS, TOF-SIMS, XPS, and XRD analyses reveal uniform lithium distribution, lithium silicate shell formation, and stable interfacial bonding between Si, Li, O, and C species. Importantly, the process is demonstrated to universally employ common and widely available lithium salts, distinguishing it from existing prelithiation methods that rely on reactive or exotic lithium precursors.

Electrochemical evaluation demonstrates that the prelithiated anodes exhibit high lithium-ion diffusion coefficients (*D*_Li_ up to 3.6 × 10^–11^ cm^2^ s^−1^), low *R*_ct_ (< 20 Ω), and an ICE exceeding 97%. Under high current densities (5 A g^−1^), the anodes deliver gravimetric capacities greater than 1700 mAh g^−1^ with > 98% capacity retention after 2000 cycles. Comparable long-term cycling stability and rate performance were also achieved in full cells paired with LFP cathodes, further validating the practical applicability of the approach.

Post-mortem analyses confirm that in situ laser-triggered prelithiation effectively mitigates continuous SEI growth and mechanical degradation, preserving electrode morphology and enabling highly stable cycling. Collectively, this methodology introduces a scalable, rapid, and low-energy paradigm for prelithiation, offering a viable pathway toward high-performance, long-life silicon anodes for next-generation lithium-ion batteries.

## Supplementary Information

Below is the link to the electronic supplementary material.

Supplementary file1 (DOCX 5814 KB)
