# Supplementary material for: Laser-Driven Single-Step Synthesis of Monolithic Prelithiated Silicon-Graphene Anodes for Ultrahigh-Performance Zero-Decay Lithium-Ion Batteries
Source: Nanomicro Lett. 2026 Jan 26;18:220. doi: 10.1007/s40820-026-02074-2 (PMC12832605; doi:10.1007/s40820-026-02074-2)
Supplement: Supplementary file 1 — Supplementary file1 (DOCX 5814 KB) [file 40820_2026_2074_MOESM1_ESM.docx]

Supporting Information for

**Laser-Driven Single-Step Synthesis of Monolithic Prelithiated Silicon-Graphene Anodes for Ultrahigh-Performance Zero-Decay Lithium-Ion Batteries**

Avinash Kothuru^1†^, Gil Daffan^2†^, Fernando Patolsky^1,2,3 *^

^1^ School of Chemistry, Faculty of Exact Sciences, Tel Aviv University, Tel Aviv 69978, Israel

^2^ Department of Materials Science and Engineering, Faculty of Engineering, Tel Aviv University, Tel Aviv 69978, Israel

^3^ Tel Aviv University Center for Nanoscience and Nanotechnology, Tel Aviv University, Tel Aviv 69978, Israel

† Avinash Kothuru and Gil Daffan contributed equally to this work.

*Corresponding author. Email: [fernando@tauex.tau.ac.il](mailto:fernando@tauex.tau.ac.il) (Fernando Patolsky)

**Supplementary Figures and Tables**

**
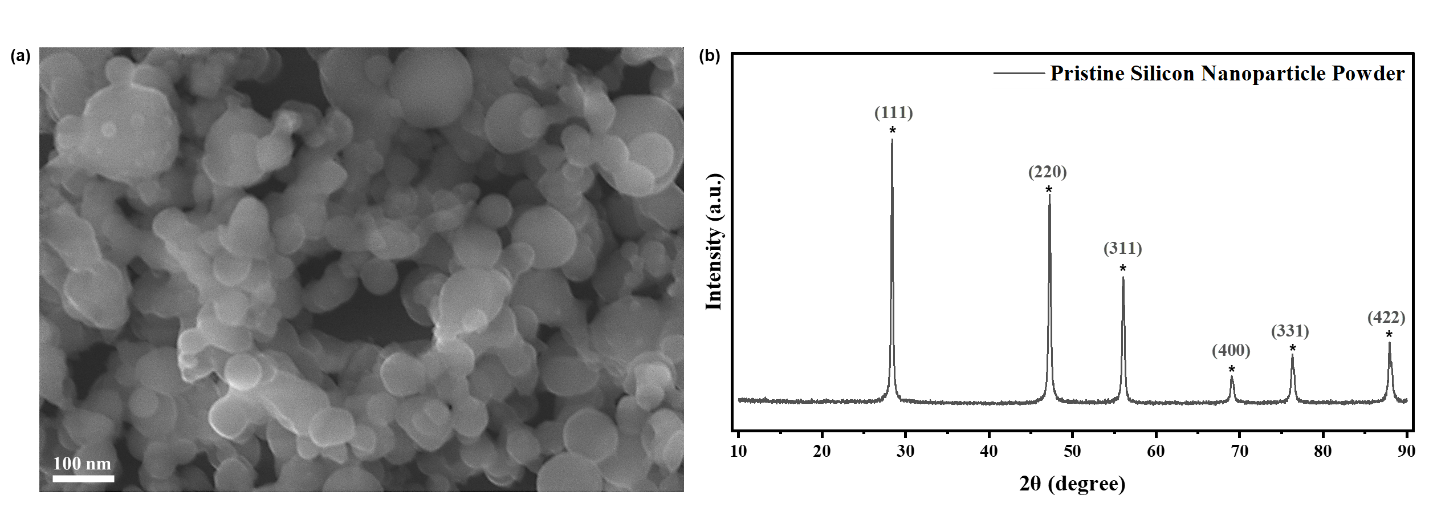
**

**Fig. S1** (**a**) High-resolution scanning electron microscopy (HR-SEM) image of pristine SiNPs, showing uniform nanoparticle morphology and surface texture prior to anode fabrication. (**b**) XRD analysis of the pristine SiNPs, showing high crystallinity before the lasing process


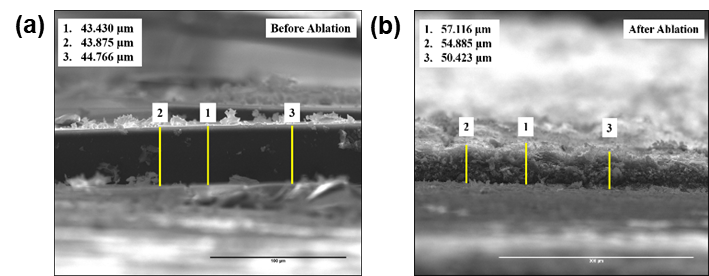


**Fig. S2** HR-SEM imaging of the prelithiated SiNP/LIG electrode cross-section, depicting electrode thickness before (**a**) and after (**b**) laser irradiation

**
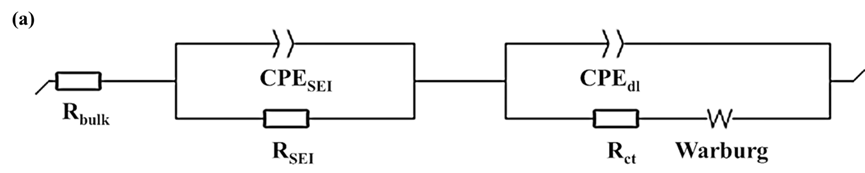
**

**Fig. S3** The equivalent circuit used for fitting the electrical impedance spectroscopy (EIS) data includes several components. The bulk resistance of the cell, referred to as R_Bulk_, corresponds to the high-frequency intercept of the first semicircle in the Nyquist plot. The solid electrolyte interphase (SEI) is modeled with a resistance (R_SEI_) and a constant phase element (CPE_SEI_). The interface between the electrode and the electrolyte is characterized by the charge transfer resistance (R_CT_) and the double-layer constant phase element (CPE_dl_). At low frequencies, impedance caused by diffusion limitations—known as Warburg impedance—appears as a line with an approximately 45-degree slope in the Nyquist plot [S1]

**
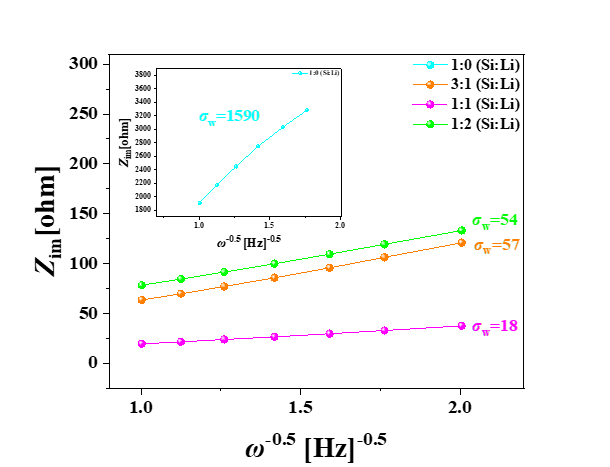
**

**Fig. S4** Slopes extracted from the low-frequency region of the EIS Nyquist plots in Fig. 4a of the manuscript, corresponding to the Warburg impedance, were calculated using equation (3) in the Methods section to analyze lithium-ion diffusion characteristics in prelithiated SiNP/LIG anodes with varying Si:LiOH ratios

**
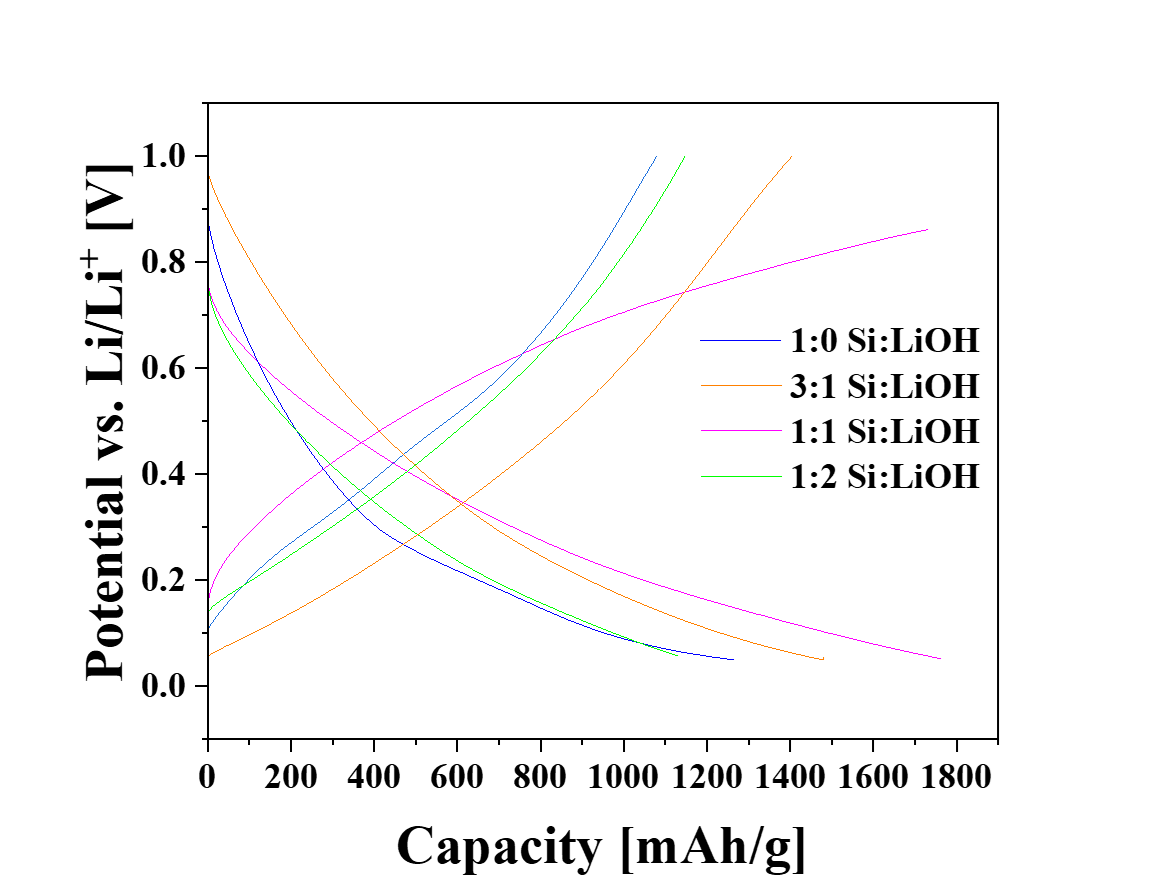
**

**Fig. S5** Voltage profiles from the initial cycles of SiNP/LIG anodes prepared with different Si:LiOH precursor ratios. The Li-free sample (1:0) exhibits an ICE of 83%, whereas all Li-containing samples show markedly higher values—94% (3:1), 97% (1:2), and 97% (1:1)—highlighting the beneficial effect of Li addition on first-cycle efficiency


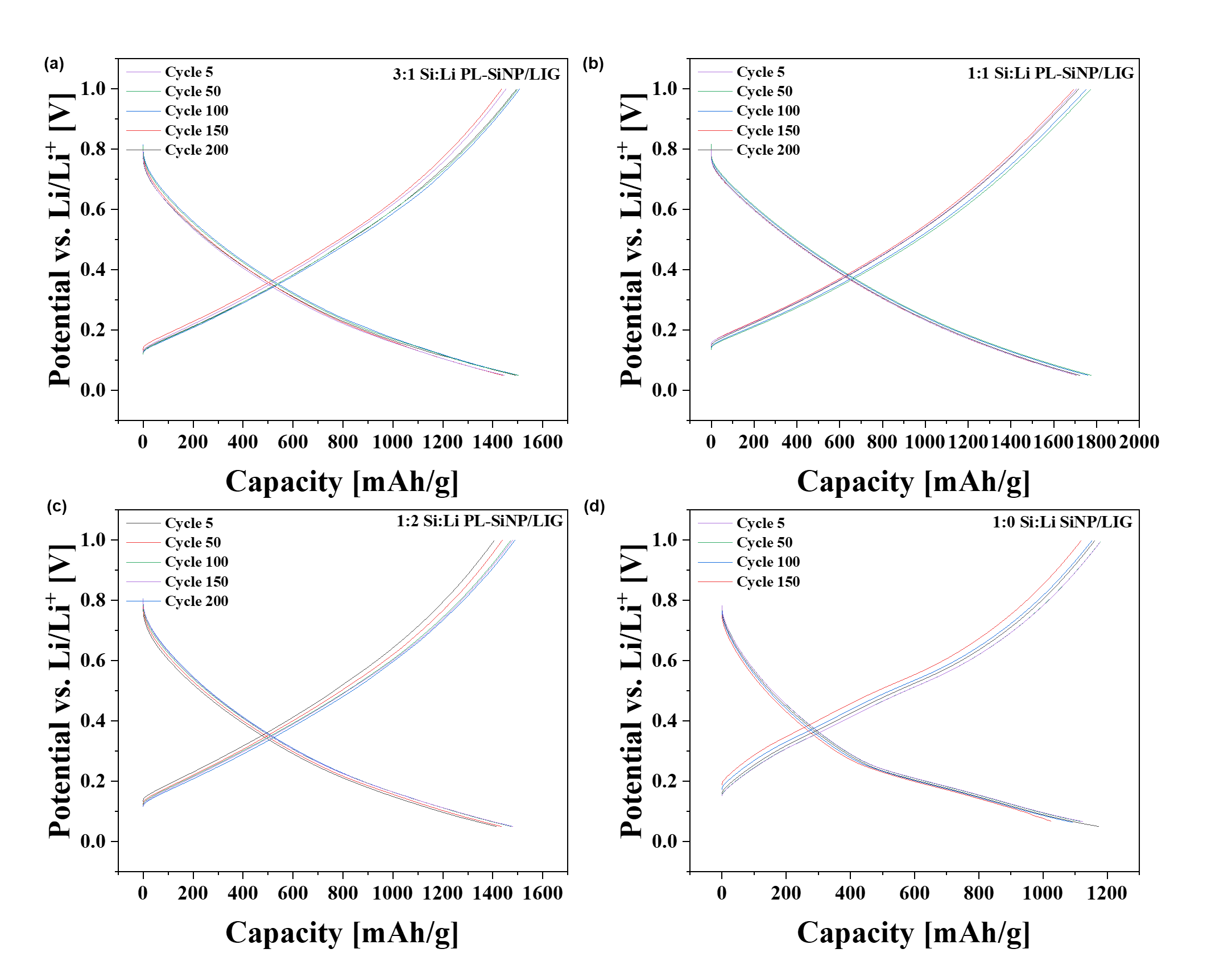


**Fig. S6.** Voltage profiles at selected cycles for prelithiated SiNP/LIG anodes prepared with different Si:LiOH precursor ratios, showing standard voltage–capacity behavior for Si-based anodes in Li-ion half-cells [S2, S3]

**
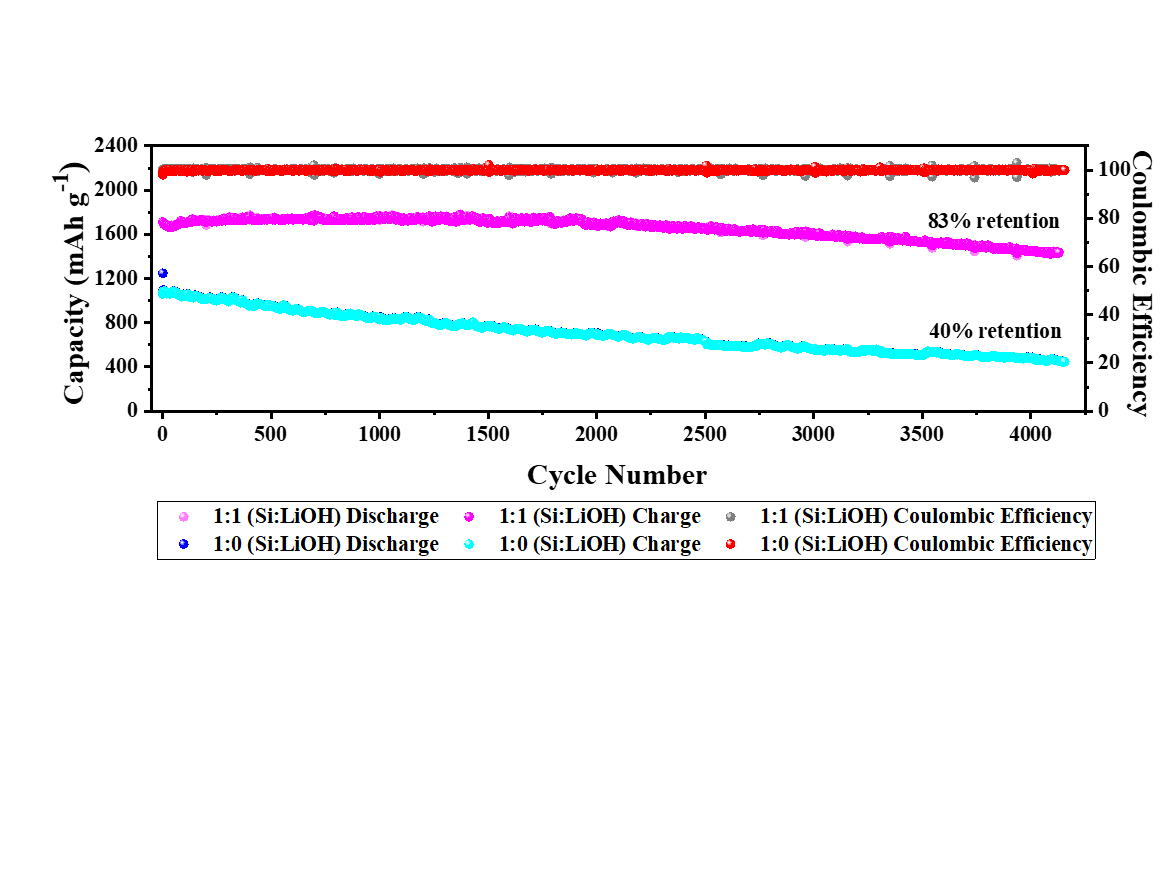
**

**Fig. S7** Galvanostatic cycling performance of non-prelithiated (turquoise) and prelithiated (magenta) SiNP/LIG anodes in lithium half-cells at a current density of 5 A/g over 4150 cycles. The prelithiated anode demonstrates exceptional long-term stability, retaining 98% of its capacity after more than 2000 cycles and 83% after 4150 cycles. In contrast, the non-prelithiated anode exhibits significant capacity degradation, retaining only ~*4*0% of its initial capacity after 4150 cycles

**
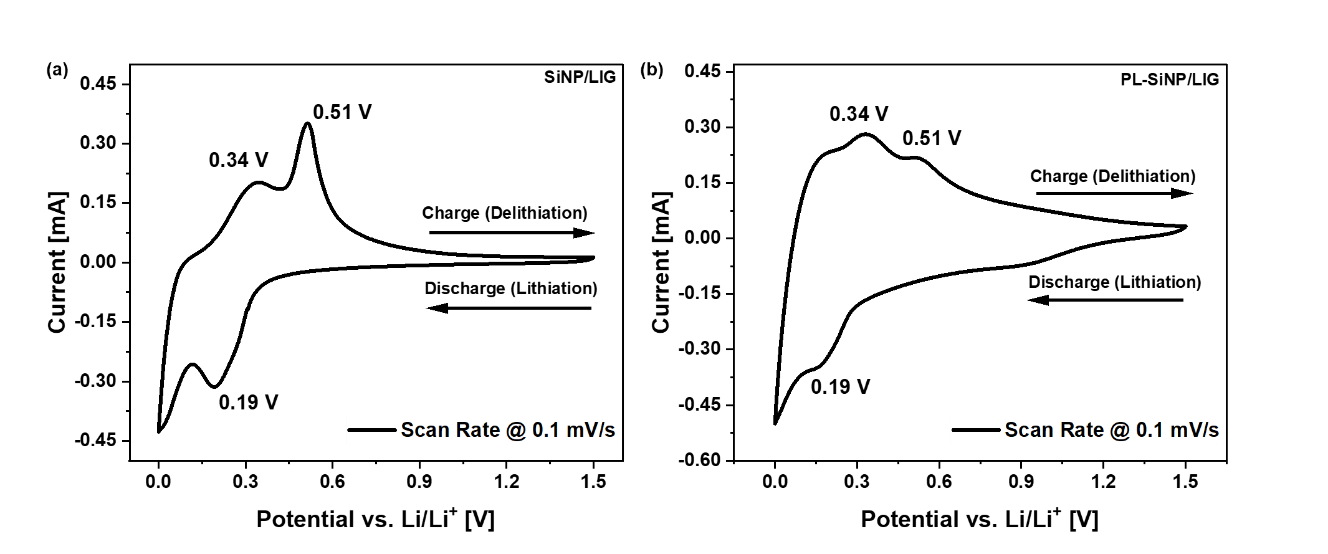
**

**Fig. S8.** Cyclic voltammetry (CV) of (a) non-prelithiated and (b) prelithiated SiNP/LIG anodes versus lithium metal in coin cells, measured between 0 and 1.5 V vs. Li/Li⁺, showing standard lithiation/delithiation peaks for silicon-based anodes in LIB half-cells [S4]

**
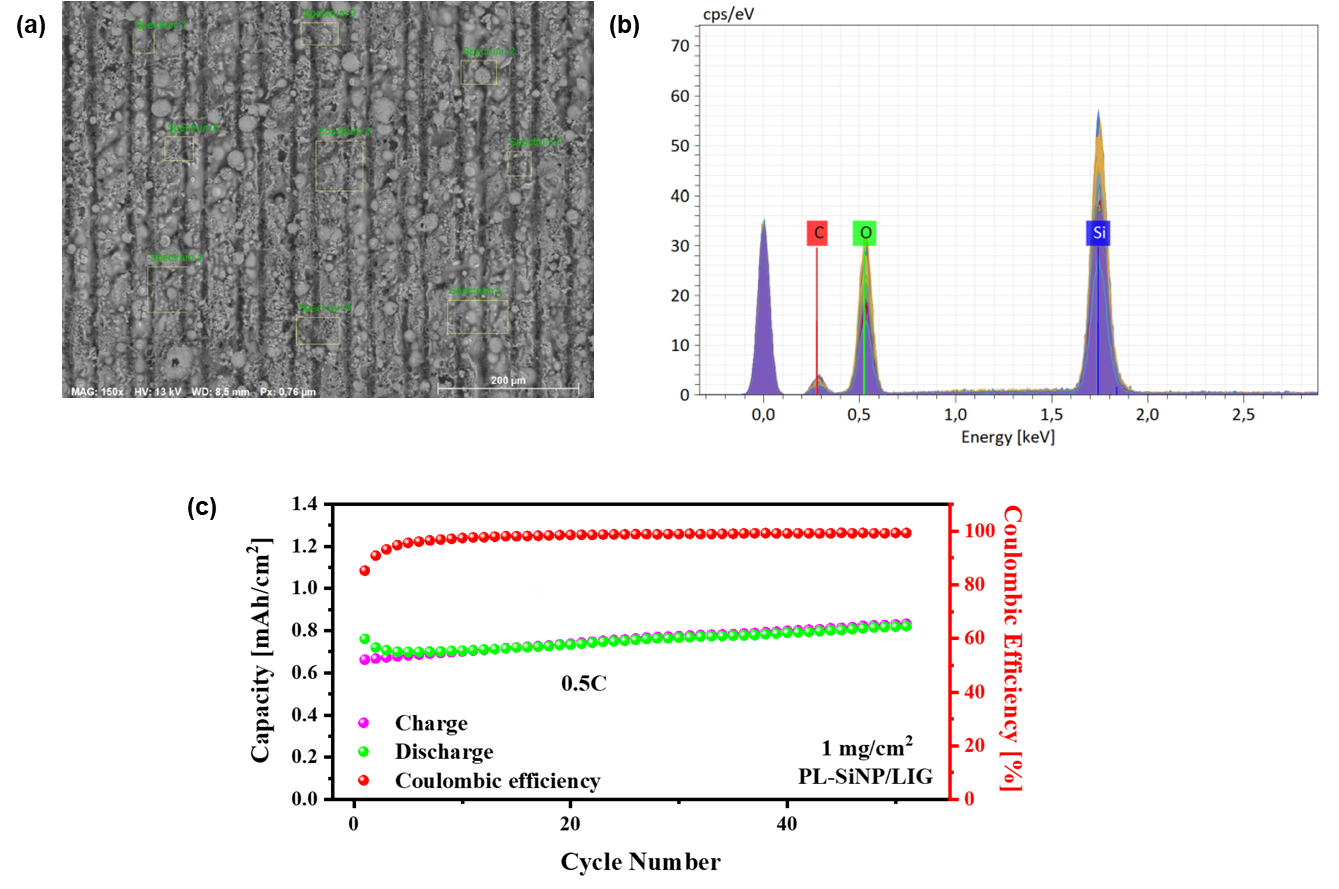
**

**Fig. S9** (**a**) HRSEM imaging of a prelithiated SiNP/LIG anode, showing selected areas (green) analyzed by energy dispersive X-ray spectroscopy (EDS) elemental analysis. (**b**) EDS spectrums of the selected areas. (**c**) Galvanostatic cycling of a prelithiated SiNP/LIG anode with a silicon loading of ~1 mg/cm*^2^* for 50 cycles at a 0.5C rate. The anode demonstrates high areal capacity and stable performance at high cycling rates with no observable degradation

**
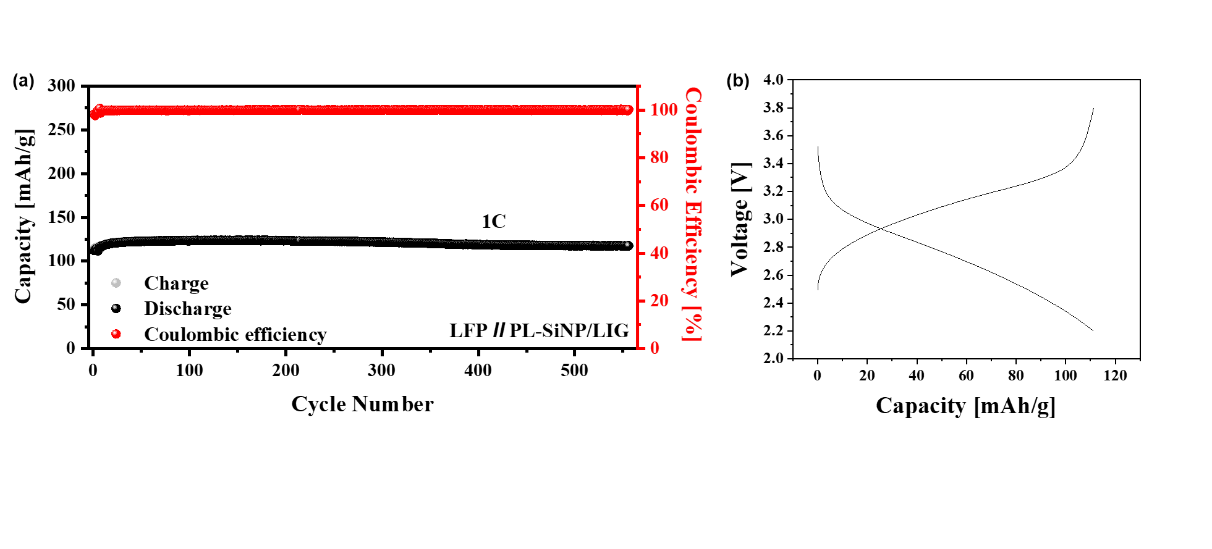
**

**Fig. S10** (**a**) Long-term galvanostatic cycling of a full cell comprising a 1 mg/cm^-2^ LiFePO_4_ (LFP) cathode and a prelithiated SiNP/LIG anode, cycled at a 1C rate between 2.8 and 3.8 V for over 500 cycles, showing stable capacity retention with no observable degradation. (**b**) Initial charge–discharge voltage profile typical of LFP-based full cells

**
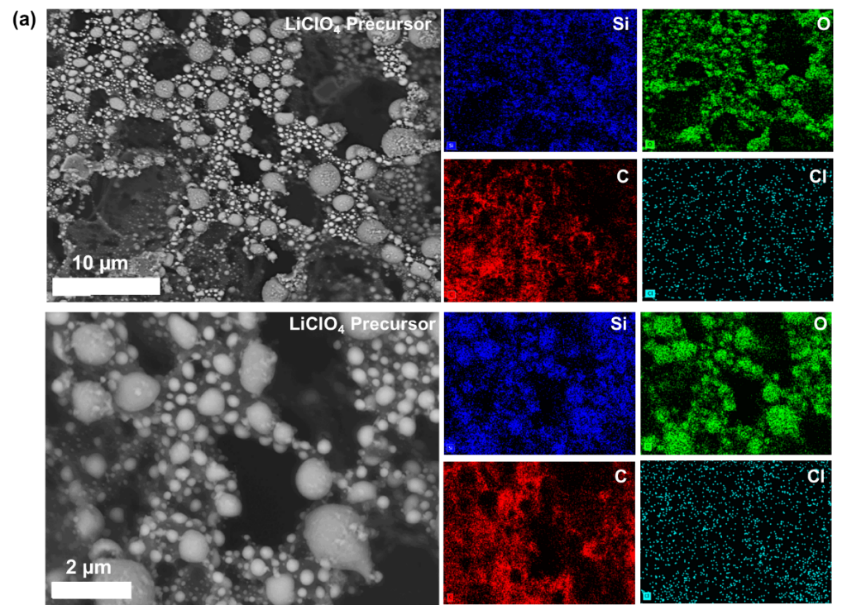
**

**Fig. S11** HRSEM and EDS elemental mapping of a prelithiated SiNP/LIG anode prepared using lithium perchlorate (LiClO*_4_*) as the lithium salt precursor


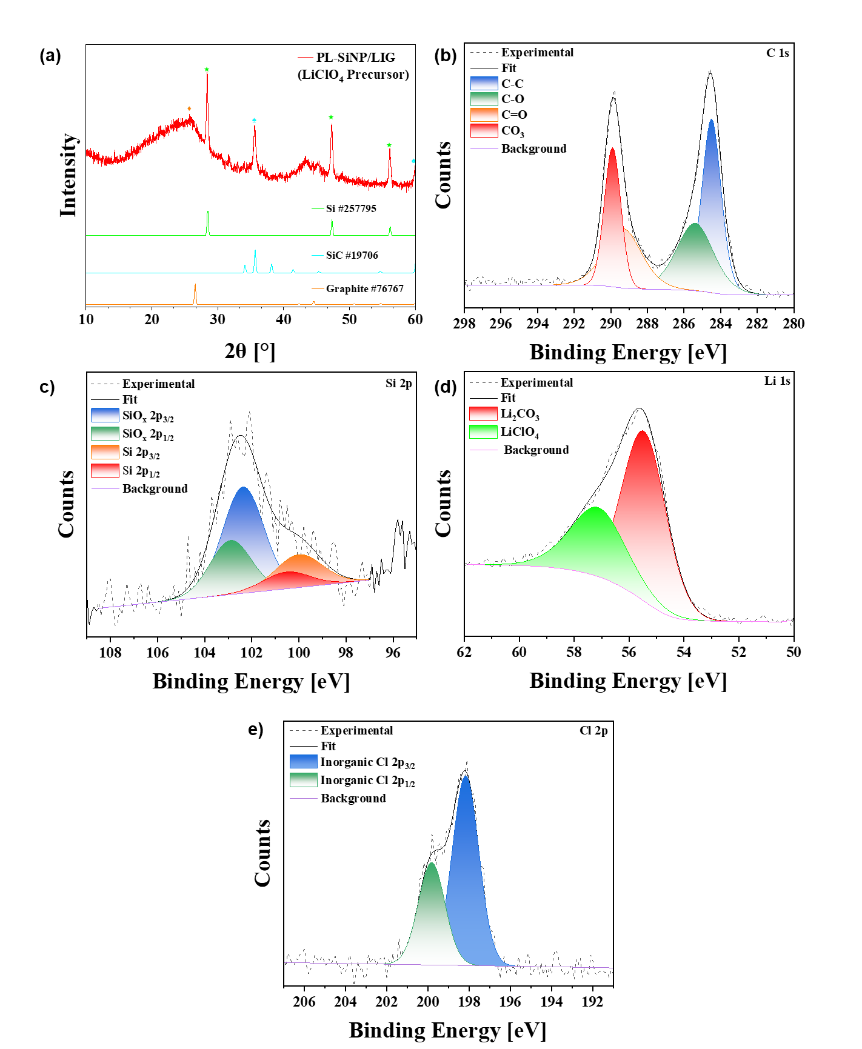


**Fig. S12** XRD (**a**) and XPS (**b-e**) analysis of a prelithiated SiNP/LIG anode prepared using lithium perchlorate (LiClO*_4_*) as the lithium salt precursor. XRD reference spectra were obtained from the ICSD database and indexed accordingly. XPS peak assignments for C 1s [S5, S6], Si 2p [S7], Li 1s [S6], and Cl 2p [S8] were performed using the NIST XPS database and cited accordingly

**
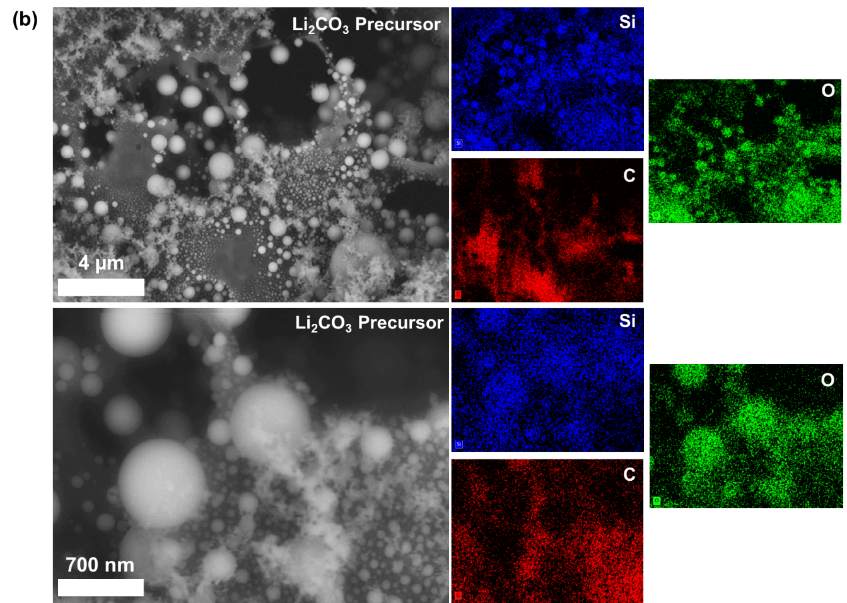
**

**Fig. S13** HRSEM and EDS imaging and elemental distribution mapping of a prelithiated SiNP/LIG anode prepared using lithium carbonate (Li*_2_*CO*_3_*) as the lithium salt precursor.


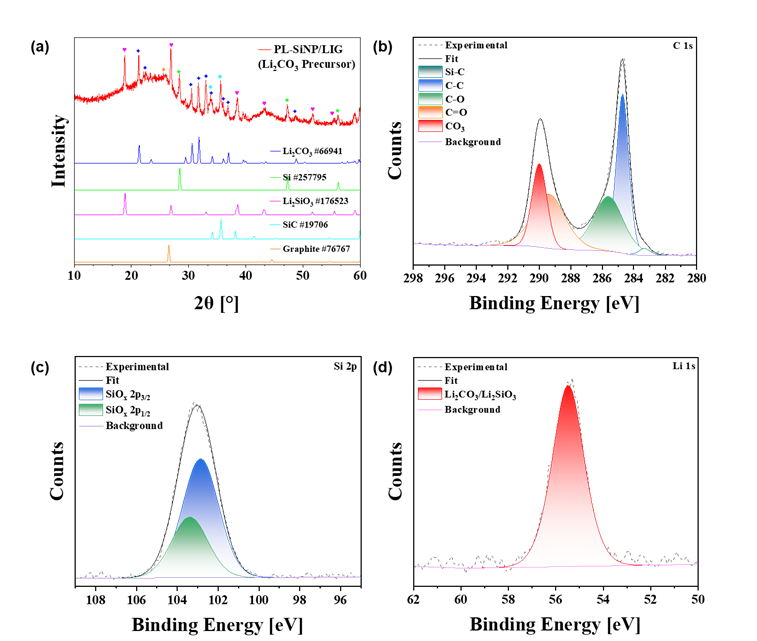


**Fig. S14** XRD (**a**) and XPS (**b-e**) analysis of a prelithiated SiNP/LIG anode prepared using lithium carbonate (Li*_2_*CO*_3_*) as the lithium salt precursor. XRD reference spectra were obtained from the ICSD database and indexed accordingly. XPS peak assignments for C 1s [S5, S6], Si 2p [S9], and Li 1s [S6, S10] were performed using the NIST XPS database and cited accordingly

**
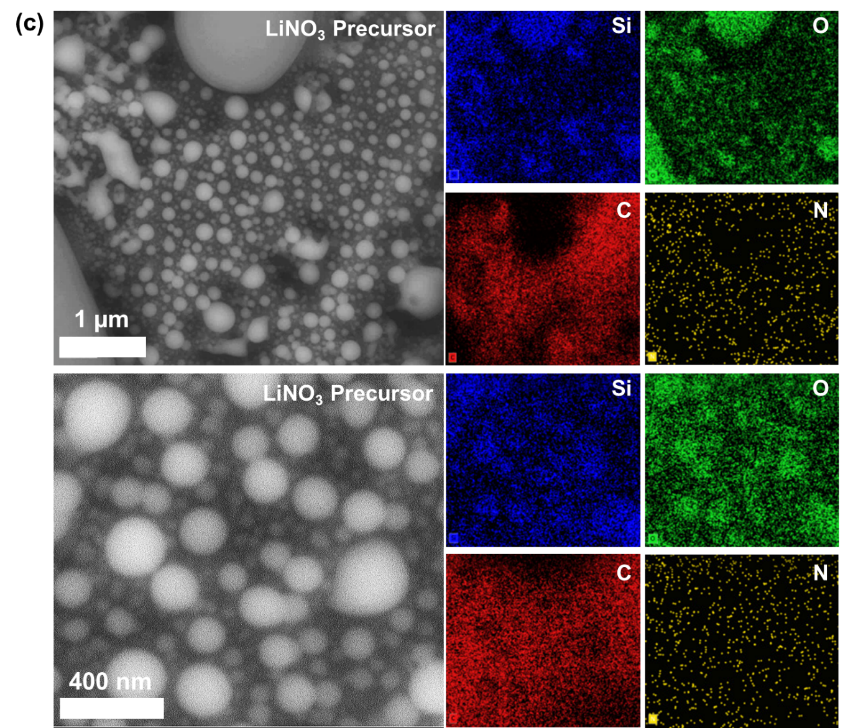
**

**Fig. S15** HRSEM and EDS imaging and elemental distribution mapping of a prelithiated SiNP/LIG anode prepared using lithium nitrate (LiNO*_3_*) as the lithium salt precursor

**
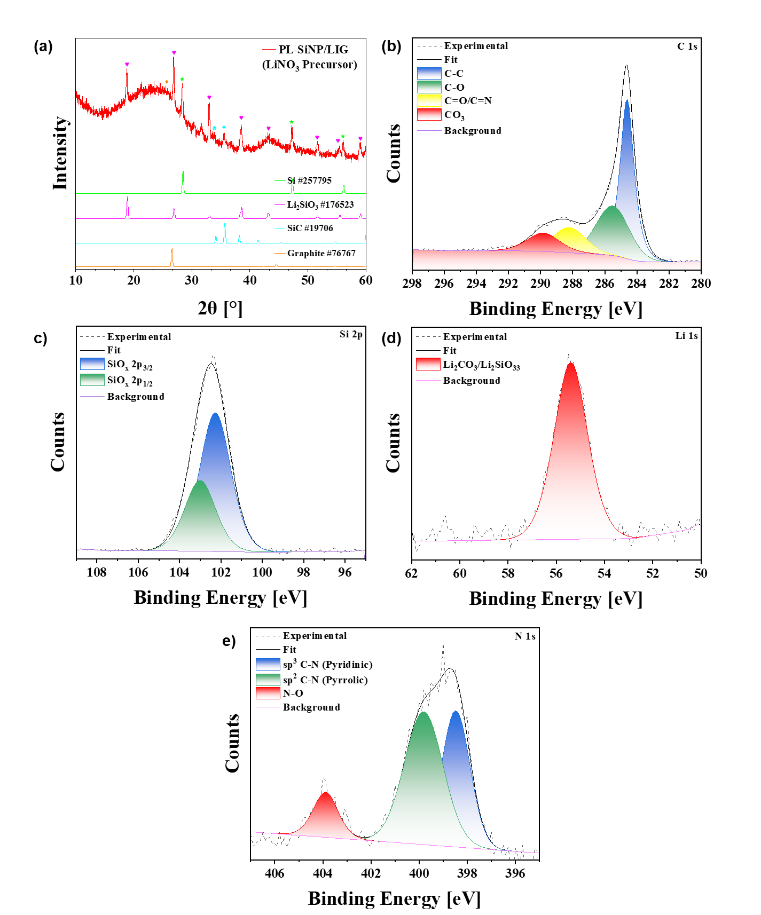
**

**Fig. S16** XRD (**a**) and XPS (**b-e**) analysis of a prelithiated SiNP/LIG anode prepared using lithium nitrate (LiNO*_3_*) as the lithium salt precursor. XRD reference spectra were obtained from the ICSD database and indexed accordingly. XPS peak assignments for C 1s [S5, S6], Si 2p [S9], Li 1s [S6, S10], and N 1s [S11]were performed using the NIST XPS database and cited accordingly

**
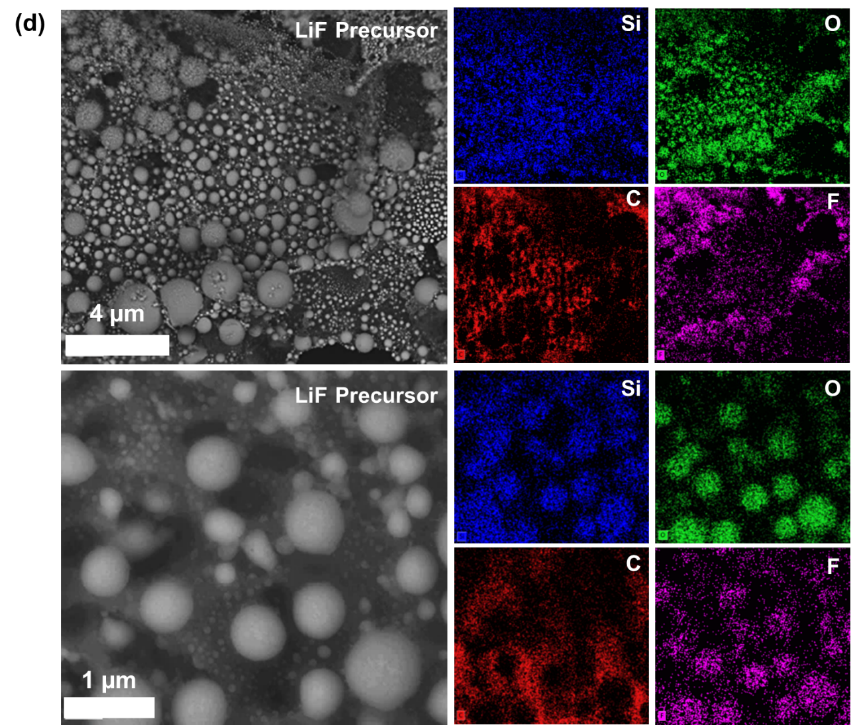
**

**Fig. S17** HRSEM and EDS imaging and elemental distribution mapping of a prelithiated SiNP/LIG anode prepared using lithium fluoride (LiF) as the lithium salt precursor


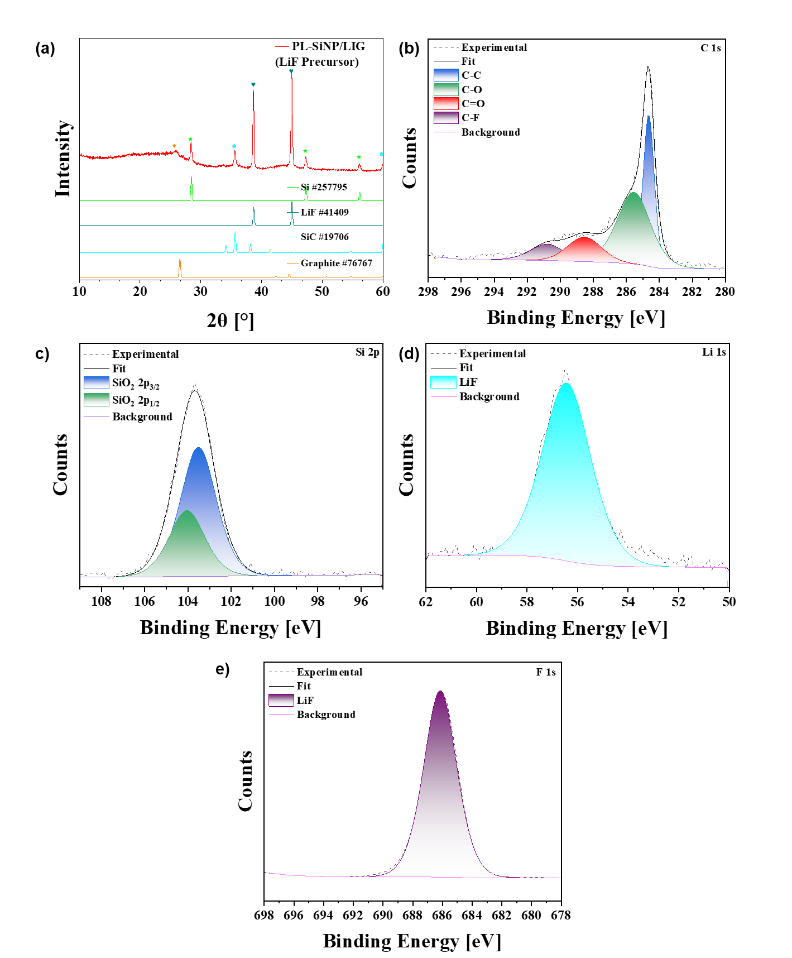


**Fig. S18** XRD (**a**) and XPS (**b-e**) analysis of a prelithiated SiNP/LIG anode prepared using lithium fluoride (LiF) as the lithium salt precursor. XRD reference spectra were obtained from the ICSD database and indexed accordingly. XPS peak assignments for C 1s [S5, S12], Si 2p [S9], Li 1s [S13], and F 1s [S13]were performed using the NIST XPS database and cited accordingly

**
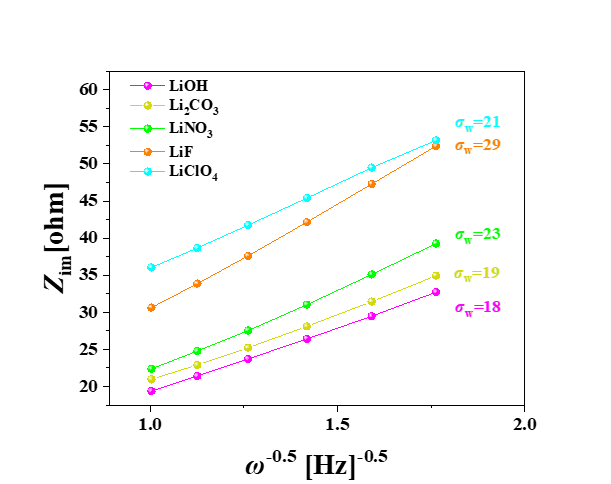
**

**Fig. S19** Slopes extracted from the low-frequency region of the EIS Nyquist plots in Fig. 5a of the manuscript, corresponding to the Warburg impedance, were calculated using equation (3) in the Methods section to analyze lithium-ion diffusion characteristics in prelithiated SiNP/LIG anodes with varying types of Li precursors

**
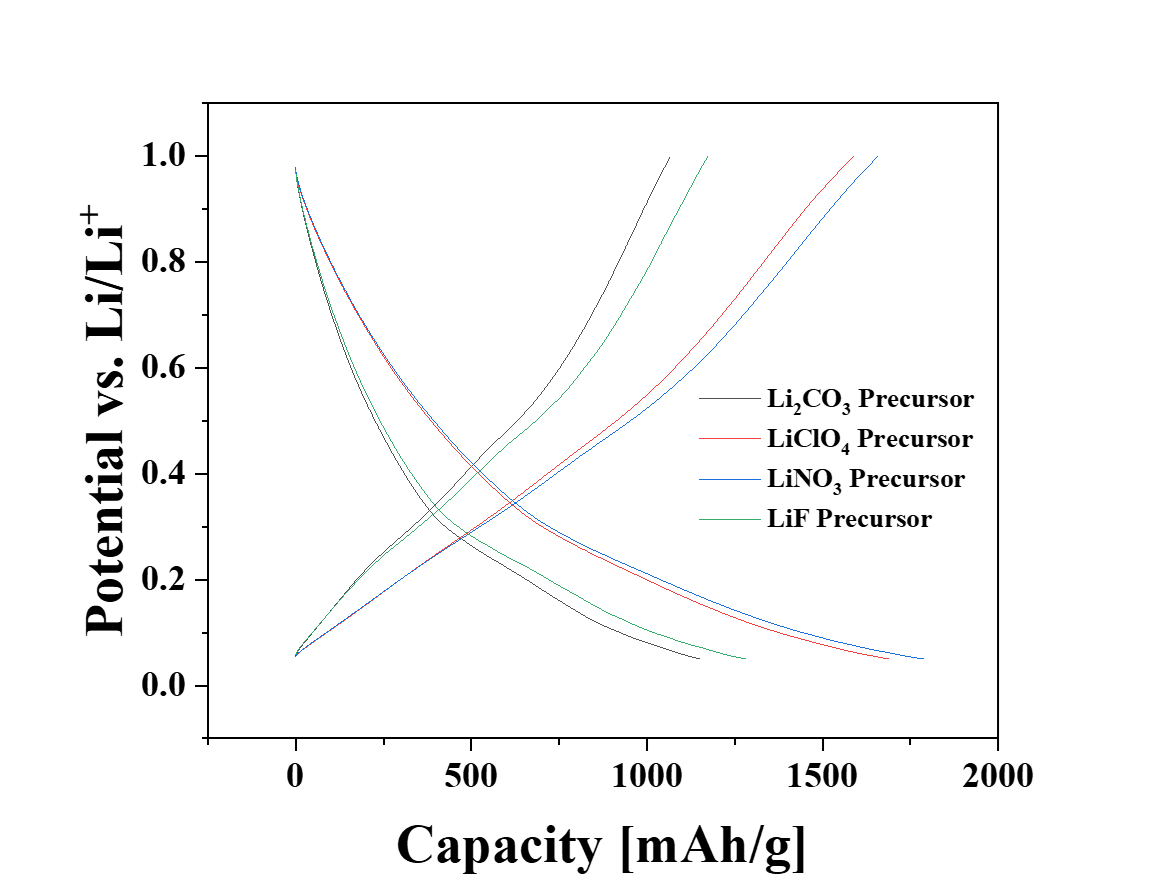
**

**Fig. S20** Voltage–capacity profiles during the initial cycles of prelithiated SiNP/LIG anodes prepared using different lithium salt precursors, showing ICEs of 92% for the carbonate precursor, 94% for the perchlorate precursor, 92% for the nitrate precursor, and 91% for the fluoride precursor

**
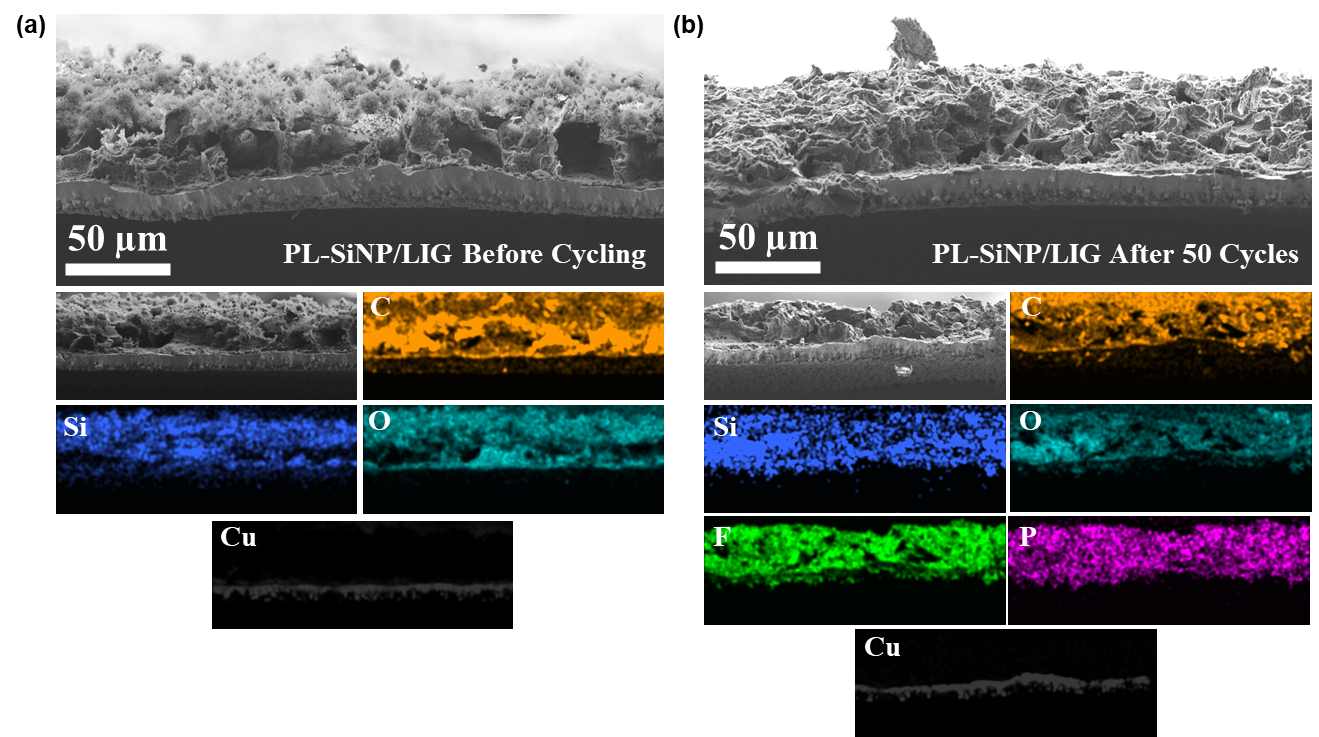
**

**Fig. S21** Cross-sectional HRSEM and EDS analysis of PL-SiNP/LIG anodes prepared using a 1:1 Si:LiOH precursor ratio before cycling (**a**) and after 50 cycles (delithiated) (**b**), showing excellent mechanical stability with no delamination, minimal volume change (∼50 µm thickness before and after cycling), and limited SEI formation


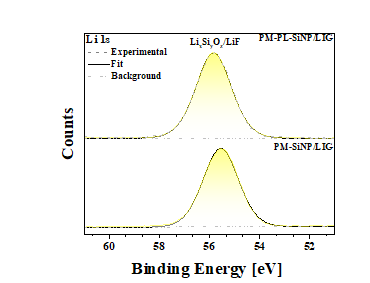


**Fig. S22** High-resolution X-ray photoelectron spectroscopy (XPS) spectra of the Li 1s region in post-mortem (PM) samples of non-prelithiated and prelithiated (PL) SiNP/LIG anodes after cycling, complementing the XPS data shown in Fig. 6e–g of the manuscript [S9, S13]

**Supplementary References**

1. L.A. Middlemiss, A.J.R. Rennie, R. Sayers, A.R. West, Characterisation of batteries by electrochemical impedance spectroscopy. Energy Rep. **6**, 232–241 (2020). <https://doi.org/10.1016/j.egyr.2020.03.029>
2. Y. Ha, T.R. Martin, S. Frisco, L. Rynearson, M.C. Schulze et al., Evaluating the effect of electrolyte additive functionalities on NMC622/Si cell performance. J. Electrochem. Soc. **169**(7), 070515 (2022). <https://doi.org/10.1149/1945-7111/ac7e75>
3. T. Vorauer, P. Kumar, C.L. Berhaut, F.F. Chamasemani, P.-H. Jouneau et al., Multi-scale quantification and modeling of aged nanostructured silicon-based composite anodes. Commun. Chem. **3**, 141 (2020). <https://doi.org/10.1038/s42004-020-00386-x>
4. B. Jerliu, E. Hüger, L. Dörrer, B.K. Seidlhofer, R. Steitz et al., Lithium insertion into silicon electrodes studied by cyclic voltammetry and operando neutron reflectometry. Phys. Chem. Chem. Phys. **20**(36), 23480–23491 (2018). <https://doi.org/10.1039/C8CP03540G>
5. B. Rui, M. Yang, L. Zhang, Y. Jia, Y. Shi et al., Reduced graphene oxide-modified biochar electrodes *via* electrophoretic deposition with high rate capability for supercapacitors. J. Appl. Electrochem. **50**(4), 407–420 (2020). <https://doi.org/10.1007/s10800-020-01397-1>
6. N. Hornsveld, B. Put, W.M.M. Kessels, P.M. Vereecken, M. Creatore, Plasma-assisted and thermal atomic layer deposition of electrochemically active Li_2_CO_3_. RSC Adv. **7**(66), 41359–41368 (2017). <https://doi.org/10.1039/c7ra07722j>
7. J.A.L. López, J.C. López, D.E.V. Valerdi, G.G. Salgado, T. Díaz-Becerril et al., Morphological, compositional, structural, and optical properties of Si-nc embedded in SiOx films. Nanoscale Res. Lett. **7**(1), 604 (2012). <https://doi.org/10.1186/1556-276X-7-604>
8. W.E. Morgan, J.R. Van Wazer, W.J. Stec, Inner-orbital photoelectron spectroscopy of the alkali metal halides, perchlorates, phosphates, and pyrophosphates. J. Am. Chem. Soc. **95**(3), 751–755 (1973). <https://doi.org/10.1021/ja00784a018>
9. K.-J. Jeong, S. Hossen, M.T. Rahman, J.S. Shim, D.-H. Lee et al., Enhancing charging efficiency with lithium silicate in silicon composite anode materials through lithiothermic reduction reaction synthesis. Adv. Mater. Technol. **9**(14), 2302055 (2024). <https://doi.org/10.1002/admt.202302055>
10. Chengxu Shenab, Rusheng Fua, Yonggao XiaORCID logo*a and Zhaoping Liu, New perspective to understand the effect of electrochemical prelithiation behaviors on silicon monoxide. RSC Adv. **8**, 14473-14478 (2018). <https://doi.org/10.1039/C8RA01917G>
11. C. Xie, L. Lin, L. Huang, Z. Wang, Z. Jiang et al., Zn-N(x) sites on N-doped carbon for aerobic oxidative cleavage and esterification of C(CO)-C bonds. Nat. Commun. **12**(1), 4823 (2021). <https://doi.org/10.1038/s41467-021-25118-0>
12. M.R. Busche, M. Weiss, T. Leichtweiss, C. Fiedler, T. Drossel et al., The Formation of the solid/liquid electrolyte interphase (SLEI) on NASICON-type glass ceramics and LiPON. Adv. Mater. Interfaces **7**(19), 2000380 (2020). <https://doi.org/10.1002/admi.202000380>
13. T. Yu, T. Zhao, N. Zhang, T. Xue, Y. Chen et al., Spatially confined LiF nanoparticles in an aligned polymer matrix as the artificial SEI layer for lithium metal anodes. Nano Lett. **23**(1), 276–282 (2023). <https://doi.org/10.1021/acs.nanolett.2c04242>
